# Anakinra treatment in critically ill COVID-19 patients: a prospective cohort study

**DOI:** 10.1186/s13054-020-03364-w

**Published:** 2020-12-10

**Authors:** Emma J. Kooistra, Nicole J. B. Waalders, Inge Grondman, Nico A. F. Janssen, Aline H. de Nooijer, Mihai G. Netea, Frank L. van de Veerdonk, Esther Ewalds, Johannes G. van der Hoeven, Matthijs Kox, Peter Pickkers, the RCI-COVID-19 Study Group, Emma J. Kooistra, Nicole J. B. Waalders, Inge Grondman, Nico A. F. Janssen, Aline H. de Nooijer, Mihai G. Netea, Frank L. van de Veerdonk, Esther Ewalds, Johannes G. van der Hoeven, Matthijs Kox, Peter Pickkers, Pleun Hemelaar, Remi Beunders, Niklas Bruse, Tim Frenzel, Jeroen Schouten, Hugo Touw, Sjef van der Velde, Hetty van der Eng, Noortje Roovers, Margreet Klop-Riehl, Jelle Gerretsen, Wout Claassen, Hidde Heesakkers, Tirsa van Schaik, Leonie Buijsse, Leo Joosten, Quirijn de Mast, Martin Jaeger, Ilse Kouijzer, Helga Dijkstra, Heidi Lemmers, Reinout van Crevel, Josephine van de Maat, Gerine Nijman, Simone Moorlag, Esther Taks, Priya Debisarun, Heiman Wertheim, Joost Hopman, Janette Rahamat-Langendoen, Chantal Bleeker-Rovers, Hans Koenen, Esther Fasse, Esther van Rijssen, Manon Kolkman, Bram van Cranenbroek, Ruben Smeets, Irma Joosten

**Affiliations:** 1 Department of Intensive Care Medicine, Radboud University Medical Center, 6500HB Nijmegen, The Netherlands; 2 Radboud Center for Infectious Diseases, Radboud University Medical Center, 6500HB Nijmegen, The Netherlands; 3 Department of Internal Medicine, Radboud University Medical Center, 6500HB Nijmegen, The Netherlands; 4 Department of Immunology and Metabolism, Life and Medical Sciences Institute (LIMES), University of Bonn, Bonn, Germany; 5 Department of Intensive Care Medicine, Bernhoven Hospital, 5406PT Uden, The Netherlands

**Keywords:** Interleukin-1 receptor antagonist protein, Coronavirus disease 2019, SARS-CoV-2, Critical care, Immunity, Cytokines

## Abstract

**Background:**

A subset of critically ill COVID-19 patients develop a hyperinflammatory state. Anakinra, a recombinant interleukin-1 receptor antagonist, is known to be effective in several hyperinflammatory diseases. We investigated the effects of anakinra on inflammatory parameters and clinical outcomes in critically ill, mechanically ventilated COVID-19 patients with clinical features of hyperinflammation.

**Methods:**

In this prospective cohort study, 21 critically ill COVID-19 patients treated with anakinra were compared to a group of standard care. Serial data of clinical inflammatory parameters and concentrations of multiple circulating cytokines were determined and aligned on start day of anakinra in the treatment group, and median start day of anakinra in the control group. Analysis was performed for day − 10 to + 10 relative to alignment day. Clinical outcomes were analyzed during 28 days. Additionally, three sensitivity analyses were performed: (1) using propensity score-matched groups, (2) selecting patients who did not receive corticosteroids, and (3) using a subset of the control group aimed to match the criteria (fever, elevated ferritin) for starting anakinra treatment.

**Results:**

Baseline patient characteristics and clinical parameters on ICU admission were similar between groups. As a consequence of bias by indication, plasma levels of aspartate aminotransferase (ASAT) (*p* = 0.0002), ferritin (*p* = 0.009), and temperature (*p* = 0.001) were significantly higher in the anakinra group on alignment day. Following treatment, no relevant differences in kinetics of circulating cytokines were observed between both groups. Decreases of clinical parameters, including temperature (*p* = 0.03), white blood cell counts (*p* = 0.02), and plasma levels of ferritin (*p* = 0.003), procalcitonin (*p* = 0.001), creatinine (*p* = 0.01), and bilirubin (*p* = 0.007), were more pronounced in the anakinra group. No differences in duration of mechanical ventilation or ICU length of stay were observed between groups. Sensitivity analyses confirmed these results.

**Conclusions:**

Anakinra is effective in reducing clinical signs of hyperinflammation in critically ill COVID-19 patients. A randomized controlled trial is warranted to draw conclusion about the effects of anakinra on clinical outcomes.

## Background

In December 2019, the severe acute respiratory syndrome coronavirus 2 (SARS-CoV-2) was identified in Wuhan, China, which led to a pandemic in 2020. The clinical spectrum of SARS-CoV-2 infection ranges from asymptomatic cases to severe viral sepsis leading to organ dysfunction and high mortality. In the absence of a vaccine, effective treatments for coronavirus disease 2019 (COVID-19) are highly warranted.

Inflammation plays a key role in the pathophysiology of COVID-19. Initial reports have shown that patients exhibit strongly elevated concentrations of several circulating pro-inflammatory cytokines and chemokines [1, 2]. Additionally, critically ill COVID-19 patients display higher plasma concentrations of pro-inflammatory cytokines than mild cases [3], suggestive of a relationship between inflammation and disease severity. A subset of severe COVID-19 patients may even develop severe hyperinflammation, showing similarities with macrophage activation syndrome (MAS), which is characterized by features such as hyperferritinemia, fever, pancytopenia, hepatobiliary dysfunction (HBD), and diffuse intravascular coagulation (DIC) [4–7]. The pro-inflammatory cytokine interleukin (IL)-1β plays a critical role in MAS through enhancing production of cytokines, including IL-1β itself, activation of endothelium with fluid extravasation, hypotension, and even death [4]. This positive feedback loop creates a vicious circle of inflammation and tissue pathology [4].

Anakinra, a recombinant IL-1 receptor antagonist (IL-1RA) which is currently used in the treatment of patients with rheumatoid arthritis, cryopyrin-associated periodic syndrome (CAPS), and Still’s disease, has been shown to be effective for the treatment of a subgroup of severe bacterial sepsis patients that demonstrate features of MAS [8]. Recently, small studies suggest that anakinra may also be effective in treating COVID-19 patients with features of a MAS-like syndrome, including persistent high fever and elevated plasma levels of ferritin. COVID-19 patients that received treatment with anakinra showed improvement of respiratory function and a decreased mortality risk compared to patients that did not receive anakinra [9–13]. Although these results are promising, limitations related to the retrospective comparison with historical control data are clear. Also, the number of treated patients remains small and mainly focussed on non-critically ill patients. Finally, data of the putative immunomodulatory effects of anakinra are limited.

In the present study, our primary objective was to investigate the effects of anakinra on the inflammatory response in critically ill COVID-19 patients with features of hyperinflammation, compared to a contemporary control group receiving standard care. The secondary objective was to explore differences in clinical outcomes between these groups. In addition, we performed three sensitivity analyses: (1) using propensity score-matched groups, (2) selecting patients who did not receive corticosteroids and (3) using a subset of the control group aimed to match the criteria (fever, elevated ferritin) for starting anakinra treatment.

## Methods

### Study design and participants

In this prospective cohort study, consecutive mechanically ventilated COVID-19 patients admitted to the intensive care unit (ICU) in the Radboud University Medical Center (Nijmegen, The Netherlands) and Bernhoven Hospital (Uden, The Netherlands) between March 11 and April 27 were screened for inclusion. COVID-19 was diagnosed by a positive SARS-CoV-2 RT-PCR test in nasopharyngeal and throat swabs and by typical chest CT-scan findings. Patients with a pre-existing immunosuppressed status or other comorbidities that could strongly influence prognosis were excluded. Indication for starting treatment with anakinra was based on clinical judgment of features of hyperinflammation (including persistent high fever and/or a high plasma level of ferritin and/or progressive organ dysfunction with no apparent reason apart from hyperinflammation). Patients who were not treated with anakinra were designated to the control group. Treatment was started with 300 mg anakinra intravenously (i.v.), followed by 100 mg i.v. every six hours. Blood sampling was carried out as part of a cohort study in critically ill COVID-19 patients, which was carried out in accordance with the applicable rules concerning the review of research ethics committees and informed consent in the Netherlands. All patients or legal representatives were informed about the details of this cohort study and could decline to participate.

All included patients who received anakinra (*n* = 21) were compared to included patients who received standard care (control group, *n* = 39). We also performed three sensitivity analyses: one in which we compared all anakinra-treated patients to a propensity score-matched control group receiving standard care (matched on baseline demographic characteristics), another in which only patients who did not receive corticosteroids were included, and a third in which the anakinra group was compared to a subgroup of the control group aimed to match criteria used to start treatment with anakinra (either fever > 38.5 °C for at least two days or high ferritin plasma levels [> 1800 µg/L]). Details are provided in Additional File 1*.*

### Data collection

Details on collection of clinical data are provided in Additional File 1. In the anakinra group, serial data were aligned on the day anakinra treatment was started, which was designated day 0. Data of the control group were aligned on the median day anakinra was started in the anakinra group (day 12 after ICU admission).

### Inflammatory parameters

The analysis methods for circulating cytokines and inflammatory proteomics are provided in Additional File 1. Body temperature, as well as circulating white blood cell counts and blood levels of ferritin, C-reactive protein (CRP), and procalcitonin were recorded between 10 days before and after alignment day (day − 10 until day 10). In case patients were actively cooled when they had a fever of > 40 °C, body temperature was imputed as 40 °C. In addition, serial values of creatinine, bilirubin, platelets, norepinephrine infusion rates, and PaO_2_/FiO_2_ (P/F) ratio were collected to calculate the sequential organ failure assessment (SOFA) score. As all patients were sedated, the Glasgow Coma Score was omitted from the SOFA score. Because a relevant proportion of patients were transferred to the regular ward at day 7 after alignment day, PaO_2_/FiO_2_ ratio and SOFA score data were not recorded. To prevent case-mix-related effects, data of these parameters are shown until day 6 after alignment day. Use of corticosteroids, remdesivir, and chloroquine was recorded between 10 days before and after alignment day.

### Clinical outcome data

Clinical outcomes (development of secondary infections, time on mechanical ventilation, ICU length of stay (LOS), and mortality) were recorded until 28 days after alignment day. Secondary infection was defined as ‘any infectious episode’ evidenced by the presence of positive culture(s) and time-stamped at the day the culture was performed. Infectious episodes were independently determined by two ICU physicians. In case of incongruency, a third ICU physician was consulted.

### Statistical analysis

Details are provided in Additional File 1. Briefly, data are displayed as median with interquartile range (IQR) or geometric mean with 95% confidence interval (CI). Between-group differences were assessed using Fisher’s exact tests, Mann–Whitney *U* tests, linear mixed effects model analysis on log-transformed data, log-rank tests, and t tests (indicated in legends).

## Results

### Patient characteristics

Seventy-eight consecutive critically ill patients with proven COVID-19 were screened. Nine patients were excluded because of a pre-existing immunocompromising condition. One patient refused participation. On alignment day, eight control patients were no longer in the ICU and were therefore excluded. Of the remaining 60 patients, 21 received anakinra (Additional File 2: Fig. 1). Median age was 63 [55–71] and 67 [59–72] years in the anakinra and control groups, respectively (*p* = 0.42). No significant differences in other patient characteristics, medical history, and laboratory and clinical parameters were present on ICU admission (Table 1). All patients required mechanical ventilation and sedation. On the alignment day, as a result of indication bias, patients in the anakinra group exhibited higher levels of ASAT (96 [67–142] vs. 64 [46–97] U/L, *p* = 0.009), and ferritin (2365 [1272–3713] vs. 1410 [713–1680] μg/L, *p* = 0.001, respectively) and a higher temperature (39.1 [38.3–40.0] °C) compared to the control group (37.8 [37.0–38.3] °C, *p* = 0.0002). No other significant differences between groups were present on alignment day (Table 1). APACHE II scores for the anakinra and control group on the day of alignment were 12 [9–13] and 10 [7–14], respectively (*p* = 0.41).

**Table 1:** Patient characteristics and clinical parameters at intensive care unit admission and on alignment day

	Anakinra (*n* = 21)	Control (*n* = 39)	*p* value
Sex, male	14 (67)	33 (85)	0.19
Age, years	63 [55–71]	67 [59–72]	0.42
Body mass index, kg/m^2^	27.7 [25.9–29.9]	26.8 [24.3–31.1]	0.50
APACHE II	15 [13–18]	16 [12–20]	0.46
Time from first COVID symptoms until ICU admission, days	13 [9–14]	10 [7–15]	0.33
Medical history			
Cardiovascular insufficiency	4 (19)	10 (26)	0.75
Hypertension	8 (38)	22 (56)	0.28
Respiratory insufficiency	1 (5)	2 (5)	1
Renal insufficiency	0 (0)	1 (3)	1
Metastatic neoplasm	2 (10)	2 (5)	0.61
Immunological insufficiency	0 (0)	1 (3)	1
Chronic obstructive pulmonary disease	0 (0)	5 (13)	0.15
Diabetes mellitus	7 (33)	7 (18)	0.21
Hematologic malignancy	1 (5)	0 (0)	0.35
Clinical parameters on admission day			
D-dimer, ng/mL	3380 [2028–18343]	2950 [1605–4610]	0.28
Creatinine, μmol/L	84 [68–96]	82 [65–110]	0.70
Alanine transaminase, U/L	47 [21–62]	39 [30–56]	0.95
Aspartate transaminase, U/L	55 [35–76]	51 [39–63]	0.96
Bilirubin, μmol/L	8 [6–13]	7 [5–14]	0.53
Lactate dehydrogenase, U/L	380 [322–493]	406 [324–507]	0.92
White blood cells, × 10^9^/L	8.2 [7.0–12.0]	9.8 [6.0–11.4]	0.95
Thrombocytes, × 10^9^/L	247 [189–324]	244 [181–325]	0.61
C-reactive protein, mg/L	254 [188–297]	198 [133–292]	0.22
Procalcitonin, μg/L	0.66 [0.18–1.39]	0.86 [0.32–2.88]	0.44
Ferritin, μg/L	1842 [1313–2767]	1439 [815–2396]	0.19
Temperature, °C	38.4 [37.8–38.9]	38.6 [37.5–39.3]	0.56
PaO_2_/FiO_2_ ratio, mmHg	138 [105–199]	139 [101–178]	0.94
SOFA score	7 [4–7]	6 [5–8]	0.27
Clinical parameters on alignment day			
D-dimer, ng/mL	4063 [2585–6285]	3918 [2349–5943]	0.70
Creatinine, μmol/L	92 [76–106]	79 [58–148]	0.55
Alanine transaminase, U/L	89 [55–119]	67 [49–114]	0.24
Aspartate transaminase, U/L	96 [67–142]	64 [46–97]	0.009
Bilirubin, μmol/L	6 [5–12]	5 [4–9]	0.18
Lactate dehydrogenase, U/L	371 [317–450]	348 [256–406]	0.11
White blood cells, × 10^9^/L	12.4 [10.4–15.9]	13.5 [11.4–16.6]	0.49
Thrombocytes, × 10^9^/L	351 [314–494]	366 [263–453]	0.34
C-reactive protein, mg/L	130 [90–237]	92 [59–170]	0.13
Procalcitonin, μg/L	0.66 [0.39–1.88]	0.48 [0.23–0.78]	0.07
Ferritin, μg/L	2365 [1272–3713]	1410 [713–1680]	0.001
Temperature, °C	39.1 [38.3–40.0]	37.8 [37.0–38.3]	0.0002
PaO_2_/FiO_2_, mmHg	188 [133–268]	157 [128–212]	0.18
SOFA score	6 [4–8]	5 [4–7]	0.75
APACHE II	12 [9–13]	10 [7–14]	0.41
Time from first COVID symptoms until alignment day, days	22 [19–27]	21 [18–26]	0.62

Data are presented as *n* (%) or median [IQR]. *p* values were calculated using Fisher’s exact tests and Mann–Whiney-*U* tests

### Inflammatory response

**Fig. 1:**
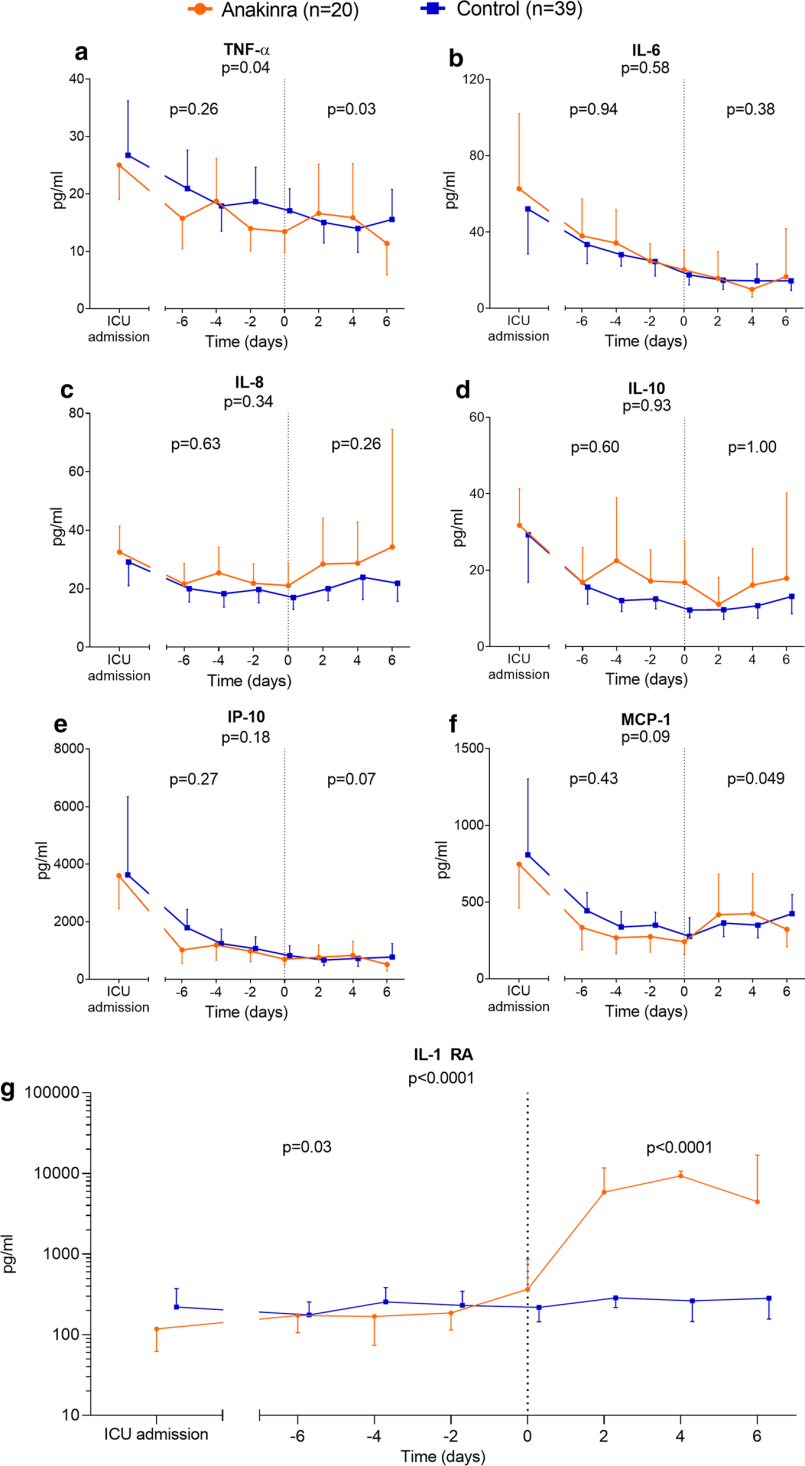
Circulating cytokine concentrations. Concentrations of circulating **a** tumor necrosis factor (TNF)-α, **b** interleukin (IL)-6, **c** IL-8, **d** IL-10, **e** interferon gamma-induced protein (IP)-10, **f** monocyte chemoattractant protein (MCP)-1, and **g** IL-1 receptor antagonist (IL-1RA) on day of intensive care unit (ICU) admission and serial data within six days pre- and post-alignment day (day 0). Data are presented as geometric mean with 95% confidence intervals and were analyzed using mixed-models analysis (time*group interaction factor) to evaluate differences between groups over time. *p* values under graph titles reflect overall between-group differences (day − 6 until day 6). Between-group *p* values for day − 6 until day 0 and day 0 until day 6 are shown on the left and right of each panel, respectively

**Fig. 2:**
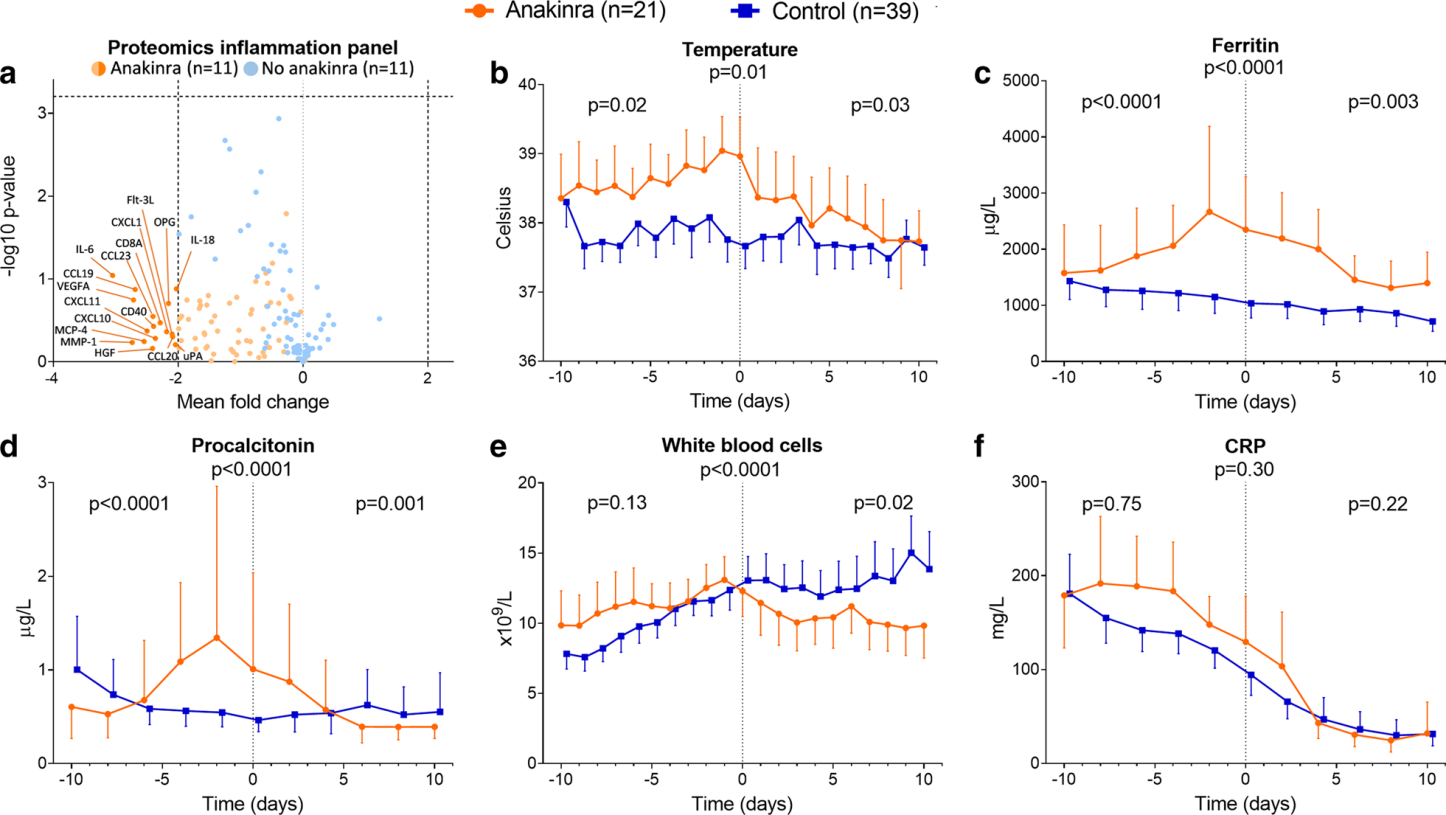
Proteomics inflammation panel and kinetics of inflammatory parameters. **a** Volcano plot of proteomics inflammation panel for 75 proteins in both groups. Mean fold change and − ^10^log(*p* value) were shown on the x-axis and y-axis, respectively. *p* values were calculated using t tests. A mean fold change of ≤ -2 or ≥ 2 was considered relevant. A − ^10^log(*p* value) > 3.204 was considered significant (p < 0.000625). **b** Body temperature and plasma levels of **c** ferritin, **d** procalcitonin, **e** white blood cell counts, and **f** C-reactive protein (CRP) over time within 10 days pre- and post-start anakinra alignment day (day 0). Data are presented as geometric mean with 95% confidence intervals and were analyzed using mixed-models analysis (time*group interaction factor) to evaluate differences between groups over time. *p* values under graph titles reflect overall between-group differences (day − 10 until day 10). Between-group *p* values for day − 10 until day 0 and day 0 until day 10 are shown on the left and right of each panel, respectively

In both groups, concentrations of all circulating cytokines decreased from ICU admission until alignment day (Fig. 1). Expectedly, administration of anakinra significantly increased circulating IL-1RA concentrations following treatment (*p* < 0.0001, Fig. 1g). After alignment day, tumor necrosis factor (TNF)-α and monocyte chemoattractant protein (MCP)-1 showed a significant difference between the anakinra and control groups (*p* = 0.03 and *p* = 0.049, respectively, Fig. 1a, f). However, a clear direction of these significant differences was absent. The kinetics of the other measured cytokines did not reveal significant differences between the anakinra group and control group (Fig. 1). Subgroups used for inflammatory proteomic analysis revealed no relevant differences in patient characteristics and clinical parameters on admission day (Additional File 3: Table 1). Of the 75 inflammatory mediators measured, 17 pro-inflammatory proteins decreased in the anakinra group post-treatment, whereas no changes were observed in the control group (Fig. 2a). Because of the large number of measured proteins and correction for multiple testing, statistical significance was not reached.

During the 10-day period before start of anakinra treatment, body temperature significantly diverted between the two groups, with higher temperatures in the anakinra group (*p* = 0.02, Fig. 2b). After anakinra treatment, temperature curves converged (*p* = 0.03, Fig. 2b). Similar effects of anakinra were observed for ferritin and procalcitonin plasma levels (Fig. 2c–d). White blood cell count decreased in the anakinra group after treatment, whereas a further increase was observed in the control group (*p* = 0.02, Fig. 2e). No significant differences in the kinetics of CRP plasma levels were present between both groups, although the decrease in CRP post-treatment appeared to be more pronounced in the anakinra group (Fig. 2f).

**Fig. 3:**
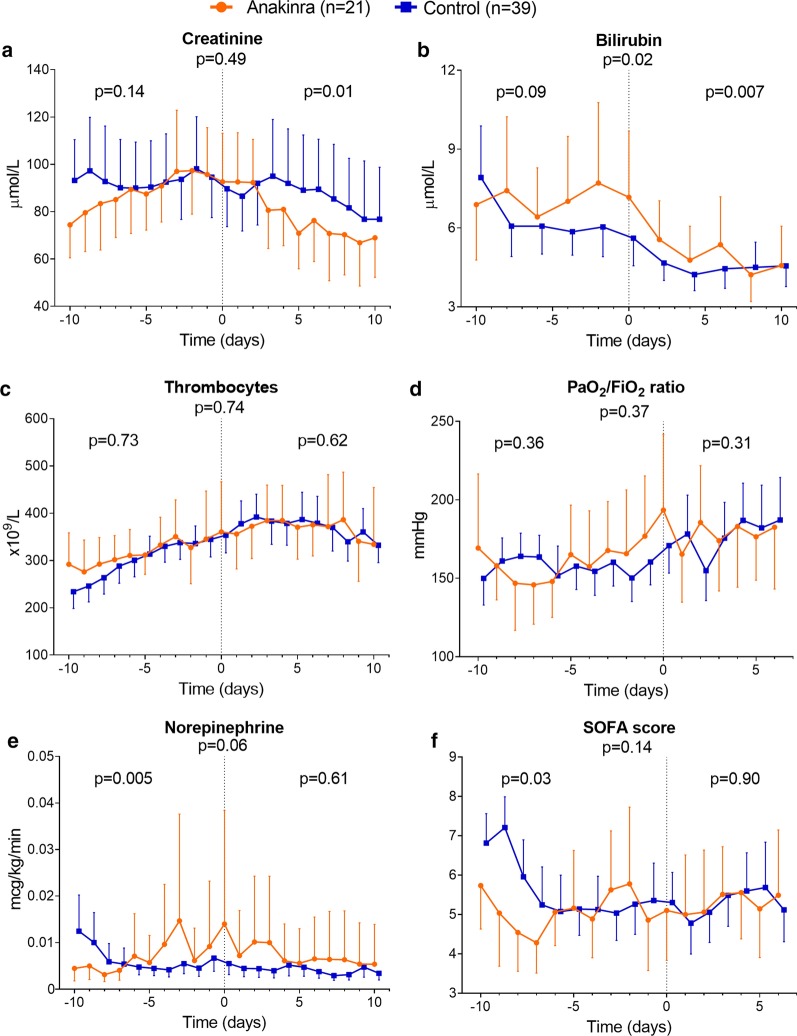
Individual parameters of sequential organ failure assessment (SOFA) score and total SOFA score. Plasma levels of **a** creatinine, **b** bilirubin, and **c** thrombocytes and **d** PaO_2_/FiO_2_ (P/F) ratio, **e** infusion rate of norepinephrine, and **f** SOFA score over time within 10 days pre- and post-alignment day (day 0). PaO_2_/FiO_2_ ratio and SOFA score were presented until day 6. Data are presented as geometric mean with 95% confidence intervals and were analyzed using mixed-models analysis (time*group interaction factor) to evaluate differences between groups over time. *p* values under graph titles reflect overall between-group differences (day − 10 until day 6 or 10). Between-group *p* values for day − 10 until day 0 and day 0 until day 6 or 10 are shown on the left and right of each panel, respectively

The decrease in plasma levels of creatinine and bilirubin was significantly more pronounced in the anakinra group compared to the control group after start of treatment (*p* = 0.01 and *p* = 0.007, respectively, Fig. 3a–b). Norepinephrine infusion rate was significantly higher in the anakinra group before treatment (*p* = 0.005, Fig. 3e), whereas no significant differences were present afterward (*p* = 0.61, Fig. 3e). No significant differences in thrombocyte counts, PaO_2_/FiO_2_ ratio, or total SOFA score were observed (Fig. 3).

The use of corticosteroids tended to be lower in the anakinra group (5%) compared to the control group (26%) before alignment day (*p* = 0.08, Additional File 4: Fig. 2a). This difference became smaller after start of treatment (anakinra group 14% and control group 28%, *p* = 0.14, Additional File 4: Fig. 2a). No differences between both groups were present pertaining to the use of remdesivir or chloroquine (Additional File 4: Fig. 2b, c).

### Clinical outcomes

**Fig. 4:**
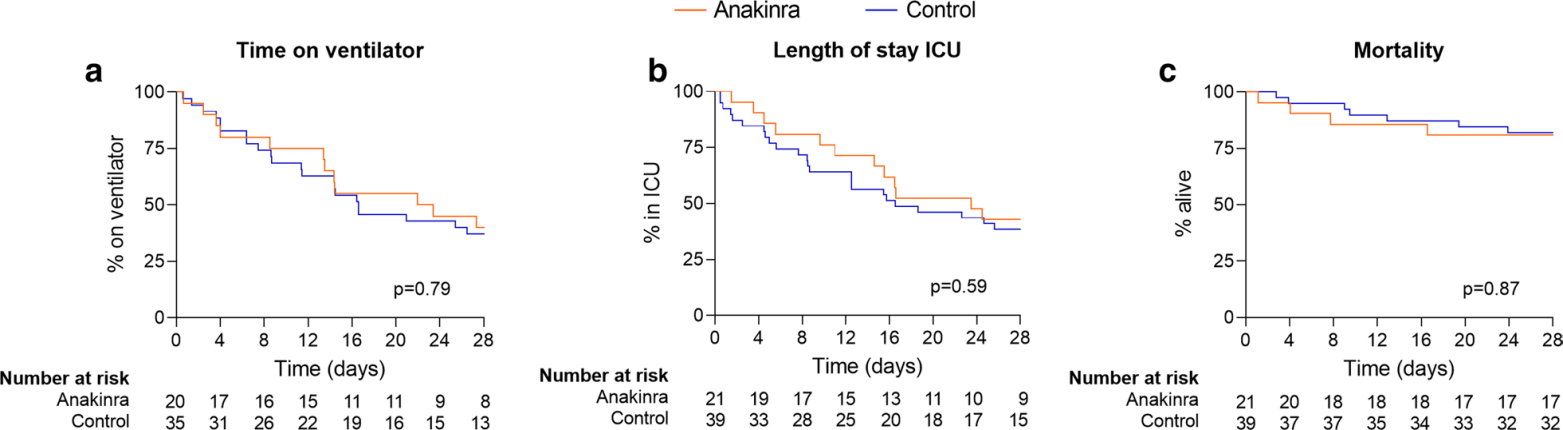
Clinical outcomes. Kaplan–Meier graphs of **a** time on mechanical ventilator, **b** length of stay in the intensive care unit (ICU), and **c** mortality. Data are presented for the first 28 days after anakinra alignment day. Patients who were no longer mechanically ventilated on alignment day were not included in time on ventilator graph. *p* values were calculated using log-rank tests. Numbers at risk on each timepoint per group are shown below graphs

A total of seven patients (33%) of the anakinra group developed a secondary infection during the first 28 days after alignment day versus nine patients (23%) of the control group (*p* = 0.54). Time on mechanical ventilation was 23 [10–29] days in the anakinra group and 17 [7–29] days in the control group (*p* = 0.79). ICU length of stay was 24 [10–29] days in the anakinra group and 17 [6–29] days in the control group (*p* = 0.59). In the anakinra group, 28-day mortality was 19% (*n* = 4) vs. 18% (*n* = 7) in the control group (*p* = 0.87). Kaplan–Meier curves are shown in Fig. 4.

### Sensitivity analyses

The propensity score-matched analysis, the analysis restricted to patients who did not receive corticosteroids, and the analysis using a subgroup of control patients aimed to match criteria to start anakinra all yielded comparable results as the primary analysis, as detailed in the additional results (Additional Files 5–21: Tables 2–4, Figs. 3–15).

## Discussion

In the present study, the effects of anakinra in critically ill COVID-19 patients with clinical features of MAS/hyperinflammation were compared to critically ill COVID-19 patients that did not display these features. Upon start of treatment, body temperature and plasma levels of ferritin and ASAT were significantly higher in the anakinra group, which supports the indications to initiate this therapy. Anakinra treatment resulted in attenuation of the clinical inflammatory response compared with the control group, including a decrease in body temperature, ferritin, white blood cell counts, and procalcitonin plasma levels. Furthermore, a more pronounced improvement in kidney and liver function was observed in the anakinra group. No significant difference was found for the overall SOFA score, likely due to limited statistical power. These findings on clinical parameters of inflammation support anti-inflammatory effects of anakinra in critically ill COVID-19 patients with features of hyperinflammation. No differences in clinical outcome parameters were observed.

Apart from IL1-RA, no relevant between-group differences in circulating IL-6 or other cytokines over time were observed. It is likely that the significant increase in plasma IL-1RA levels after start of anakinra treatment is due to detection of the drug compound itself, as anakinra is recombinant IL-1RA and was administered intravenously. Although anakinra blocks IL-1β signaling, this cytokine is of little theragnostic use, because of its short half-life which renders it undetectable in the circulation in most cases [14]. Based on our current findings, circulating concentrations of other cytokines appear to be a poor reflection of the immunomodulating effects of anakinra as well, and are therefore not likely to be useful as biomarkers to start treatment and/or monitor the effects of anakinra in critically ill COVID-19 patients.

Our study did not show an obvious clinical benefit of the anti-inflammatory effects of anakinra treatment. However, it is of paramount importance to realize that this was not a randomized controlled trial, and that patients in the anakinra and control group may not have had the same prognosis at the moment of alignment. Patients that were treated with anakinra clearly exhibited signs of hyperinflammation, while this was not the case for patients in the control group. It is well known that hyperinflammation/MAS in sepsis patients is associated with impaired clinical prognosis [15]. In accordance, in the post hoc analysis of the anakinra trial in bacterial sepsis patients, the presence of MAS was associated with a mortality of 65% [8]. Treatment with anakinra in this subgroup of MAS patients was associated with a significantly reduced mortality of 35%, which was similar to the 30% mortality observed in sepsis patients without MAS in the same trial. In line with these results, clinical outcomes of COVID-19 patients treated with anakinra in the present study were comparable with those of the control group, who did not display hyperinflammation and may have had a better a priori prognosis. Nevertheless, our study design does not allow for conclusions of possible clinical benefits of anakinra treatment.

While the use of corticosteroids between the two arms of the study did not reach statistical significance, it was fivefold more prevalent in the group without anakinra before the start of the intervention, and remained twice as high after start of anakinra therapy. Because corticosteroids can affect inflammatory parameters and were recently reported to decrease mortality in COVID-19 [16, 17], we performed a sensitivity analysis in patients who did not receive corticosteroids during the study period. This analysis yielded similar results to our primary analysis, ruling out corticosteroids as an important confounder.

The absence of significant respiratory improvement and/or reduced mortality in the anakinra group contrasts previous findings [9–12]. This might be due to the fact that we included critically ill mechanically ventilated COVID-19 patients, whereas previous studies were performed in less severe cases with either no ventilatory support or non-invasive ventilation [9–12]. Possibly, earlier or preventive treatment with anakinra may be more effective and/or disease severity is of relevance for the therapeutic efficacy of anakinra. Also, circulating concentrations of all measured cytokines in our study were markedly lower on alignment day than on ICU admission in both groups. It might be speculated that anakinra is more effective in an earlier, more pro-inflammatory stage of COVID-19 pre-ICU admission, during which higher concentrations of cytokines are present. Furthermore, the previously reported clinical improvements in COVID-19 patients treated with anakinra may be due to notable differences in patient characteristics between treatment groups in earlier studies. For example, anakinra was started in case of mild hyperinflammation (e.g., ferritin ≥ 900 ng/ml or CRP ≥ 100 mg/L, or both), whereas the control group had substantially higher levels of both ferritin and CRP compared to the anakinra group [9] or were compared to historical data of bacterial sepsis patients with MAS in the absence of a COVID-19 control group [10]. These issues might have confounded the clinical outcome results demonstrated in these studies. So, in the absence of a prospective controlled trial randomizing patients that fulfill a hyperinflammatory profile, we feel it is too early to judge any possible clinical effects and to decide what would be the optimal timing to start treatment with anakinra in critically ill COVID-19 patients.

This study has several limitations. First, as inclusion criteria were applied to start treatment with anakinra and these criteria were not present in the control group, bias by indication was clearly present. This difference is likely of importance for the prognosis of the patients, so as a consequence, no direct link can be deducted between the use of anakinra and the clinical results. Nevertheless, we did use a contemporary control group, which was not the case in previous studies [9–12]. Also, we performed propensity score matching based on patient characteristics which yielded similar results. Additionally, aimed to address this possible bias by indication, an additional sensitivity analysis using a subgroup of control patients who partially met the criteria to start anakinra treatment was performed. Although this matching was not perfect (as the presence of fever was shorter, ferritin was somewhat less elevated and patients had no signs of progressing organ failure), this group was better matched to the anakinra group than the control group used for the main analyses. This additional sensitivity analysis also showed a more pronounced decrease in clinical inflammatory markers in the anakinra group compared to the control group. Of relevance, and confirming the main analyses, no trends toward differences in clinical outcome parameters were found. Second, limited statistical power because of the relatively small number of patients preclude conclusions related to the presence or absence of efficacy of anakinra on clinical outcome parameters. Nevertheless, the inflammation-related clinical endpoints show a clear signal in accordance with the mechanism of action of anakinra and kidney and liver function appear to improve with anakinra therapy.

## Conclusions

Anakinra reduces clinical inflammatory parameters in severe COVID-19 patients with features of hyperinflammation. Results of this study, including three sensitivity analyses, do not indicate efficacy of anakinra on clinical outcome parameters. A larger multicenter randomized controlled trial is warranted to demonstrate the presence/absence of clinical efficacy of anakinra in severely ill COVID-19 patients.

## Supplementary information

**Additional file 1**. Additional description of study methods.

**Additional file 2: Figure 1**. Patient flowchart.

**Additional file 3: Table 1**. Description of data: Characteristics and clinical parameters at ICU admission and on alignment day for patients included in the inflammatory proteomics analysis. Data are presented as *n* (%) or median [IQR]. *P* values were calculated using Fisher’s exact tests and Mann–Whitney *U* tests.

**Additional file 4: Figure 2**. Description of data: Use of medication. Differences of use of **(a)** corticosteroids, **(b)** remdesivir, and **(c)** chloroquine between anakinra group and control group during 10 days before and 10 days after alignment day (day 0). *P *values were calculated using Fisher’s exact tests.

**Additional file 5**. Description of additional results.

**Additional file 6: Table 2**. Description of data: Patient characteristics and clinical parameters at ICU admission and on alignment day in propensity score-matched groups. Data are presented as *n* (%) or median [IQR]. *P* values were calculated using Fisher’s exact tests and Mann–Whitney *U* tests.

**Additional file 7: Table 3**. Description of data: Patient characteristics and clinical parameters at ICU admission and on alignment day for the subgroup analysis in patients who received no corticosteroids. Data are presented as *n* (%) or median [IQR]. *P *values were calculated using Fisher’s exact tests and Mann–Whitney *U* tests.

**Additional file 8: Table 4**. Description of data: Patient characteristics and clinical parameters at ICU admission and on alignment day for the subgroup analysis with control patients who partially met the criteria to start anakinra treatment. Data are presented as *n* (%) or median [IQR]. *P* values were calculated using Fisher’s exact tests and Mann–Whitney *U* tests.

**Additional file 9: Figure 3**. Description of data: Circulating cytokine concentrations in propensity score-matched groups. Concentrations of circulating **(a)** tumor necrosis factor (TNF)-α, **(b)** interleukin (IL)-6, **(c)** IL-8, **(d)** IL-10, **(e)** interferon gamma-induced protein (IP)-10, **(f)** monocyte chemoattractant protein (MCP)-1, and **(g)** IL-1 receptor antagonist (IL-1RA) on day of intensive care unit (ICU) admission and serial data within 6 days pre- and post-alignment day (day 0). Data are presented as geometric mean with 95% confidence intervals and were analyzed using mixed-models analysis (time*group interaction factor) to evaluate differences between groups over time. *p* values under graph titles reflect overall between-group differences (day − 6 until day 6). Between-group *p* values for day − 6 until day 0 and day 0 until day 6 are shown on the left and right of each panel, respectively.

**Additional file 10: Figure 4**. Description of data: Inflammation parameters over time in propensity score-matched groups. **(a)** Body temperature and plasma levels of **(b)** ferritin, **(c)** procalcitonin, **(d)** white blood cell counts, and **(e)** C-reactive protein (CRP) over time within 10 days pre- and post-alignment day (day 0) in propensity scored matched groups (*n* = 21 both). Data are presented as geometric mean with 95% confidence intervals and were analyzed using mixed-models analysis (time*group interaction factor) to evaluate differences between groups over time. *P* values under graph titles reflect overall between-group differences (day − 10 until day 10). Between-group *p* values for day − 10 until day 0 and day 0 until day 10 are shown on the left and right of each panel, respectively.

**Additional file 11: Figure 5**. Description of data: Individual parameters of sequential organ failure assessment (SOFA) score and total SOFA score in propensity score-matched groups. Plasma concentrations of **(a)** creatinine, **(b)** bilirubin, and **(c)** thrombocytes and **(d)** PaO_2_/FiO_2_ (P/F)-ratio, **(e)** infusion rate of norepinephrine, and **(f)** SOFA score over time within 10 days pre- and post-alignment day (day 0) in propensity score-matched groups (*n* = 21 both). PaO_2_/FiO_2_ ratio and SOFA score were presented until day 6. Data are presented as geometric mean with 95% confidence intervals and were analyzed using mixed-models analysis (time*group interaction factor) to evaluate differences between groups over time. *P *values under graph titles reflect overall between-group differences (day − 10 until day 6 or 10). Between-group *p* values for day − 10 until day 0 and day 0 until day 6 or 10 are shown on the left and right of each panel, respectively.

**Additional file 12: Figure 6**. Description of data: Use of medication in propensity score-matched groups. Differences of use of **(a)** corticosteroids, **(b)** remdesivir, and **(c)** chloroquine between anakinra group and control group during 10 days before and 10 days after alignment day (day 0). *p* values were calculated using Fisher’s exact tests.

**Additional file 13: Figure 7**. Description of data: Clinical outcomes in propensity score-matched groups. Kaplan–Meier graphs of **(a)** time on mechanical ventilator, **(b)** length of stay in the intensive care unit (ICU), and **(c)** mortality for propensity score-matched groups. Data are presented for the first 28 days after anakinra alignment day. Patients who were no longer mechanically ventilated on alignment day were not included in time on ventilator graph. *P* values were calculated using log-rank tests. Numbers at risk on each timepoint per group are shown below graphs.

**Additional file 14: Figure 8**. Description of data: Circulating cytokine concentrations for the subgroup analysis in patients who did not receive corticosteroids. Concentrations of circulating **(a)** tumor necrosis factor (TNF)-α, **(b)** interleukin (IL)-6, **(c)** IL-8, **(d)** IL-10, **(e)** interferon gamma-induced protein (IP)-10, **(f)** monocyte chemoattractant protein (MCP)-1, and **(g)** IL-1 receptor antagonist (IL-1RA) on day of intensive care unit (ICU) admission and serial data within 6 days pre- and post-alignment day (day 0). Data are presented as geometric mean with 95% confidence intervals and were analyzed using mixed-models analysis (time*group interaction factor) to evaluate differences between groups over time. *P* values under graph titles reflect overall between-group differences (day − 6 until day 6). Between-group *p* values for day − 6 until day 0 and day 0 until day 6 are shown on the left and right of each panel, respectively.

**Additional file 15: Figure 9**. Description of data: Inflammation parameters over time for the subgroup analysis in patients who did not receive corticosteroids. **(a)** Body temperature and plasma levels of **(b)** ferritin, **(c)** procalcitonin, **(d)** white blood cell counts, and **(e)** C-reactive protein (CRP) over time within 10 days pre- and post-alignment day (day 0). Data are presented as geometric mean with 95% confidence intervals and were analyzed using mixed-models analysis (time*group interaction factor) to evaluate differences between groups over time. *P* values under graph titles reflect overall between-group differences (day − 10 until day 10). Between-group *p* values for day − 10 until day 0 and day 0 until day 10 are shown on the left and right of each panel, respectively.

**Additional file 16: Figure 10**. Description of data: Individual parameters of sequential organ failure assessment (SOFA) score and total SOFA score for the subgroup analysis in patients who did not receive corticosteroids. Plasma concentrations of **(a)** creatinine, **(b)** bilirubin, and **(c)** thrombocytes and **(d)** PaO_2_/FiO_2_ (P/F)-ratio, **(e)** infusion rate of norepinephrine, and **(f)** SOFA score over time within 10 days pre- and post-alignment day (day 0). PaO_2_/FiO_2_ ratio and SOFA score were presented until day 6. Data are presented as geometric mean with 95% confidence intervals and were analyzed using mixed-models analysis (time*group interaction factor) to evaluate differences between groups over time. *P* values under graph titles reflect overall between-group differences (day − 10 until day 6 or 10). Between-group *p* values for day − 10 until day 0 and day 0 until day 6 or 10 are shown on the left and right of each panel, respectively.

**Additional file 17: Figure 11**. Description of data: Clinical outcomes for the subgroup analysis in patients who did not receive corticosteroids. Kaplan–Meier graphs of **(a)** time on mechanical ventilator, **(b)** length of stay in the intensive care unit (ICU), and **(c)** mortality. Data are presented for the first 28 days after anakinra alignment day. Patients who were no longer mechanically ventilated on alignment day were not included in time on ventilator graph. *p* values were calculated using log-rank tests. Numbers at risk on each timepoint per group are shown below graphs.

**Additional file 18: Figure 12**. Description of data: Circulating cytokine concentrations for the subgroup analysis with control patients who partially met the criteria to start anakinra treatment. Concentrations of circulating **(a)** tumor necrosis factor (TNF)-α, **(b)** interleukin (IL)-6, **(c)** IL-8, **(d)** IL-10, **(e)** interferon gamma-induced protein (IP)-10, **(f)** monocyte chemoattractant protein (MCP)-1, and **(g)** IL-1 receptor antagonist (IL-1RA) on day of intensive care unit (ICU) admission and serial data within four days pre- and 6 days post-alignment day (day 0). Data are presented as geometric mean with 95% confidence intervals and were analyzed using mixed-models analysis (time*group interaction factor) to evaluate differences between groups over time. *p* values under graph titles reflect overall between-group differences (day − 6 until day 6). Between-group *p* values for day − 6 until day 0 and day 0 until day 6 are shown on the left and right of each panel, respectively.

**Additional file 19: Figure 13**. Description of data: Inflammation parameters over time for the subgroup analysis with control patients who partially met the criteria to start anakinra treatment. **(a)** Body temperature and plasma levels of **(b)** ferritin, **(c)** procalcitonin, **(d)** white blood cell counts, and **(e)** C-reactive protein (CRP) over time within 10 days pre- and post-alignment day (day 0). Data are presented as geometric mean with 95% confidence intervals and were analyzed using mixed-models analysis (time*group interaction factor) to evaluate differences between groups over time. *P* values under graph titles reflect overall between-group differences (day − 10 until day 10). Between-group *p* values for day − 10 until day 0 and day 0 until day 10 are shown on the left and right of each panel, respectively.

**Additional file 20: Figure 14**. Description of data: Individual parameters of sequential organ failure assessment (SOFA) score and total SOFA score for the subgroup with control patients who partially met the criteria to start anakinra treatment. Plasma concentrations of **(a)** creatinine, **(b)** bilirubin, and **(c)** thrombocytes and **(d)** PaO_2_/FiO_2_ (P/F)-ratio, **(e)** infusion rate of norepinephrine, and **(f)** SOFA score over time within 10 days pre- and post-alignment day (day 0). PaO_2_/FiO_2_ ratio and SOFA score were presented until day 6. Data are presented as geometric mean with 95% confidence intervals and were analyzed using mixed-models analysis (time*group interaction factor) to evaluate differences between groups over time. *P* values under graph titles reflect overall between-group differences (day − 10 until day 6 or 10). Between-group *p* values for day − 10 until day 0 and day 0 until day 6 or 10 are shown on the left and right of each panel, respectively.

**Additional file 21: Figure 15**. Description of data: Clinical outcomes for the subgroup with control patients who partially met the criteria to start anakinra treatment. Kaplan–Meier graphs of **(a)** time on mechanical ventilator, **(b)** length of stay in the intensive care unit (ICU), and **(c)** mortality. Data are presented for the first 28 days after anakinra alignment day. Patients who were no longer mechanically ventilated on alignment day were not included in time on ventilator graph. *p* values were calculated using log-rank tests. Numbers at risk on each timepoint per group are shown below graphs.

## Data Availability

The datasets generated during and/or analyzed during the current study are available from the corresponding author on reasonable request.

## Abbreviations

CAPS: Cryopyrin-associated periodic syndrome
CI: Confidence interval
COVID-19: Coronavirus disease 2019
CRP: C-reactive protein
DIC: Diffuse intravascular coagulation
GCP: Good clinical practice
HBD: Hepatobiliary dysfunction
ICU: Intensive care unit
IL: Interleukin
IL-1RA: Interleukin-1 receptor antagonist
IP: Interferon gamma-induced protein
IQR: Interquartile ranges
i.v.: Intravenously
LOS: Length of stay
MCP: Monocyte chemoattractant protein
MAS: Macrophage activation syndrome
P/F: PaO_2_/FiO_2_
SARS-CoV-2: Severe acute respiratory syndrome coronavirus 2
SOFA: Sequential organ failure assessment
TNF: Tumor necrosis factor
