# Supplementary material for: Anakinra treatment in critically ill COVID-19 patients: a prospective cohort study
Source: Crit Care. 2020 Dec 10;24:688. doi: 10.1186/s13054-020-03364-w (PMC7726611; doi:10.1186/s13054-020-03364-w)
Supplement: Supplementary file 1 — Additional file 1. Additional description of study methods. [file 13054_2020_3364_MOESM1_ESM.docx]

**Additional file 1: Additional methods**

**Additional methods**

**Sensitivity analyses**

To correct for possible baseline differences in demographic characteristics, we also compared all anakinra-treated patients to a propensity score-matched control group receiving standard care (n=21). Propensity score matching was performed using the MatchIt package in R-studio v3.6.2 (http://www.r-project.org). The nearest neighbor method was employed using the following variables: age, sex, APACHE II score, body mass index (BMI) and medical history. Data of a patient of the control group was aligned on the day anakinra was started in his/her respective matched patient from the anakinra group.

Several patients included in this study were treated with corticosteroids for (suspected) pulmonary fibrosis, with a start dose of 25-100 mg i.v. twice daily. Because corticosteroids can modulate inflammatory parameters and possibly clinical outcome, a subgroup analysis was performed in which only patients who did not receive corticosteroids were included.

Aimed to address possible bias by indication, a third sensitivity analysis was performed using a subgroup of the control group including patients who partially met the criteria to start treatment with anakinra (either fever >38.5^o^C for at least two days or high ferritin plasma levels [>1800 µg/L]). Data of the control group was aligned on the first day of the fever episode or high ferritin plasma levels (median: day 6 of ICU stay). Because alignment day of the control group was earlier during stay in ICU, data of this sensitivity analysis are shown from day -4 onwards.

**Data collection**

Clinical data were collected from the electronic patient files (EPIC, EPIC Systems Corporation, Verona, Wisconsin, USA or NEXUS/PDMS, Nieuwegein, the Netherlands) and recorded in the good clinical practice (GCP)-compliant data management system Castor (Castor EDC, Amsterdam, the Netherlands).

**Plasma cytokines**

A baseline blood sample was obtained within the first 48 hours following ICU admission and serial samples were collected every other day within six days pre- and post-alignment day. Ethylenediaminetetraacetic acid (EDTA)-anticoagulated blood was centrifuged (2000g, 10 min, 4 ^o^C), after which plasma was stored at -80 ^o^C until analysis. Concentrations of tumor necrosis factor (TNF)-α, interleukin (IL)-6, IL-8, IL-10, interferon gamma-induced protein (IP)-10, monocyte chemoattractant protein (MCP)-1, and IL-1 receptor antagonist (IL-1RA) were determined in one batch using a Luminex assay (Milliplex, Millipore, Billerica, USA). The lower detection limit was 3.2 pg/mL for all cytokines.

**Inflammatory proteomics**

A total of 92 circulating inflammatory proteins were determined in 11 randomly sampled anakinra patients and 11 random patients of the control groups within three days before and after the alignment day using a multiplex proximity extension assay (PEA, inflammation panel, Olink Proteomics AB, Uppsala, Sweden). Proteins are expressed on a log2-scale as normalized protein expression (NPX) values, which were normalized using bridging samples to correct for batch variation. A quality control was performed per sample by Olink Proteomics. A sample that deviated < 0.3 NPX from the median passed the quality control. Proteins which did not pass the quality control or were detected in less than 80% of the samples were excluded from analysis. This led to exclusion of 17 proteins, resulting in analysis of 75 proteins in both groups.

**Statistical analysis**

Variables that were not measured daily were binned into bins spanning two or three days, depending on the frequency of measurements, using a custom script made in R-studio v3.6.2 (https://www.r-project.org).https://www.r-project.org). If more than one value was present in a two- or three-day period, the mean value was used. Because of the relatively small group size, normality was not assumed. Baseline characteristics and differences in clinical parameters on ICU admission and alignment day were analyzed using Fisher’s exact tests and Mann-Whitney-U tests. Between-group differences over time of clinical measurements and concentrations of circulating cytokines were analyzed using linear mixed effects model analysis on log-transformed data. Three mixed model analysis were performed for each variable: the overall between-group differences (day -10 until day 10), differences pre-alignment day (day -10 until day 0), and differences post-alignment (day 0 until day 10, representing the treatment effect). Differences in use of corticosteroids, remdesivir, and chloroquine, and differences in the proportion of patients who developed a secondary infection were tested using Fisher’s exact test. Time on mechanical ventilation, ICU LOS, and mortality were analyzed using log-rank tests from alignment day onwards. Patients who died in the hospital or those who were still in the ICU and/or receiving mechanical ventilation on day 28 were censored at day 29 for the analysis of time on mechanical ventilation and ICU LOS. For the mortality analysis, patients who were discharged alive from the hospital or were still in the ICU or hospital on day 28 were censored at day 29.

Proteomics data were binned into one value per protein before alignment day and one value after alignment day for each patient using the aforementioned R-script. Differences between these two values were analyzed within the anakinra and control group using t-tests. To correct for multiple testing, a p-value of <0.000625 (0.05/80 proteins) was considered as statistically significant. Fold changes (after vs. before alignment day) of ≤ -2 or ≥ 2 were considered relevant. Statistical analysis was performed using SPSS 25 (IBM) and GraphPad Prism 8 software (GraphPad Software).
