# Supplementary figures and images for: Anakinra treatment in critically ill COVID-19 patients: a prospective cohort study
Source: Crit Care. 2020 Dec 10;24:688. doi: 10.1186/s13054-020-03364-w (PMC7726611; doi:10.1186/s13054-020-03364-w)

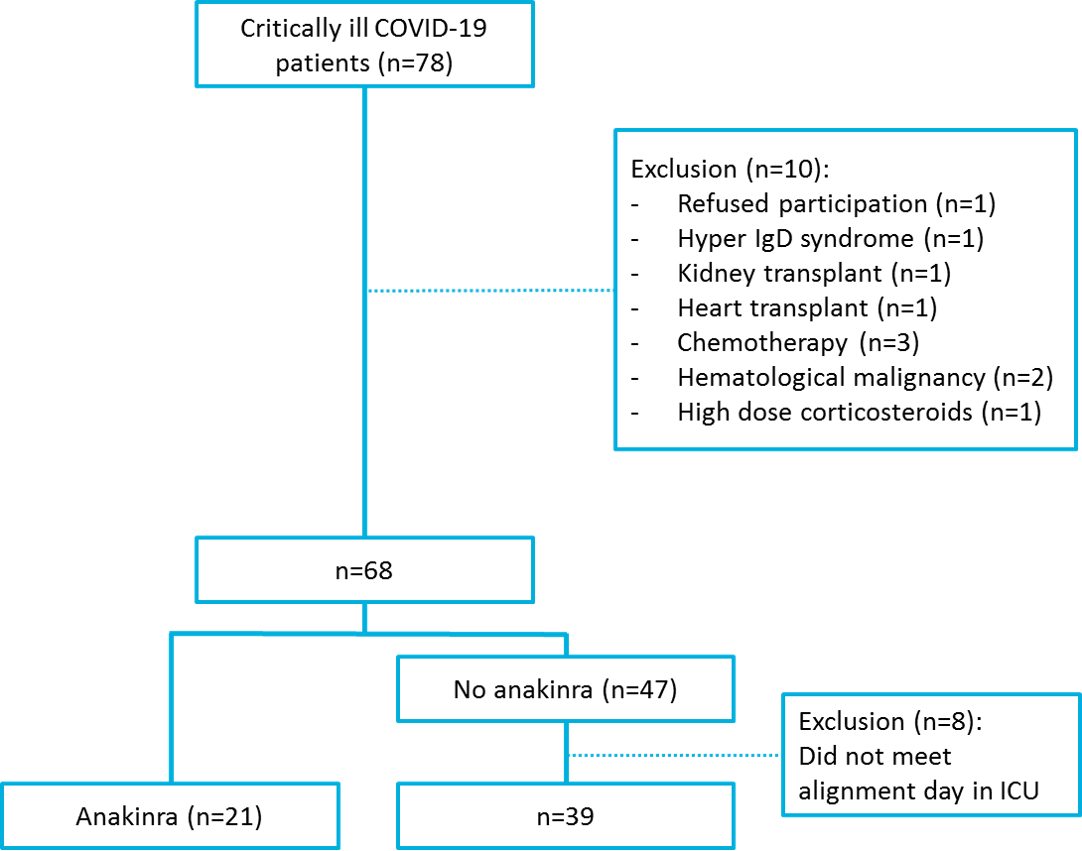

Supplement: Supplementary file 2 — Additional file 2: Figure 1. Patient flowchart. [file 13054_2020_3364_MOESM2_ESM.tif]

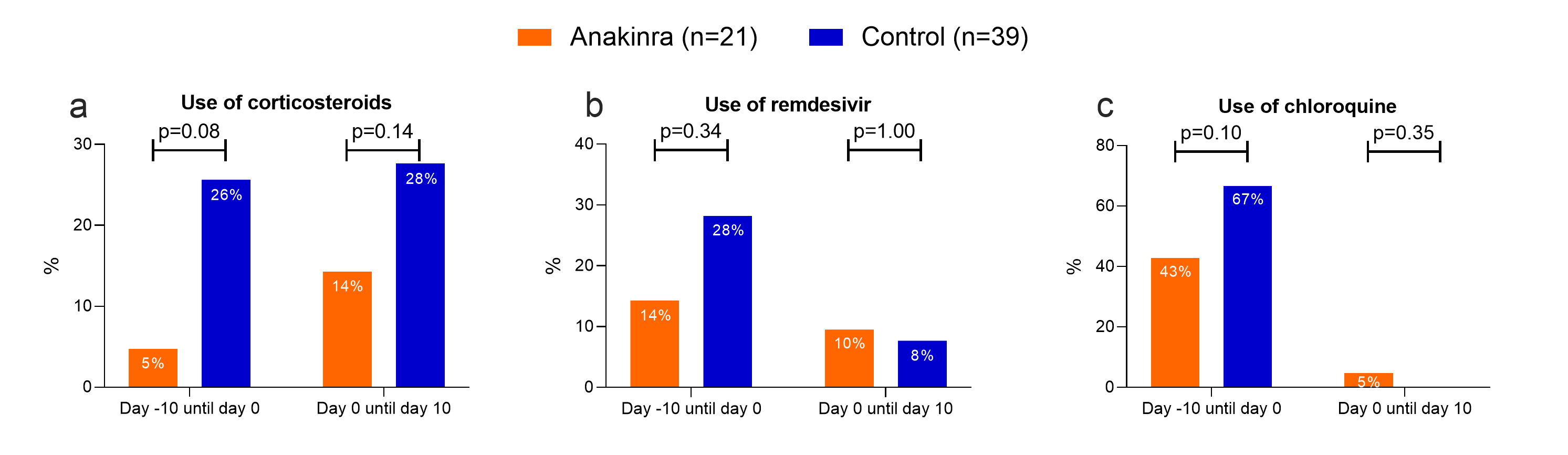

Supplement: Supplementary file 4 — Additional file 4: Figure 2. Description of data: Use of medication. Differences of use of (a) corticosteroids, (b) remdesivir, and (c) chloroquine between anakinra group and control group during 10 days before and 10 days after alignment day (day 0). P values were calculated using Fisher’s exact tests. [file 13054_2020_3364_MOESM4_ESM.tif]

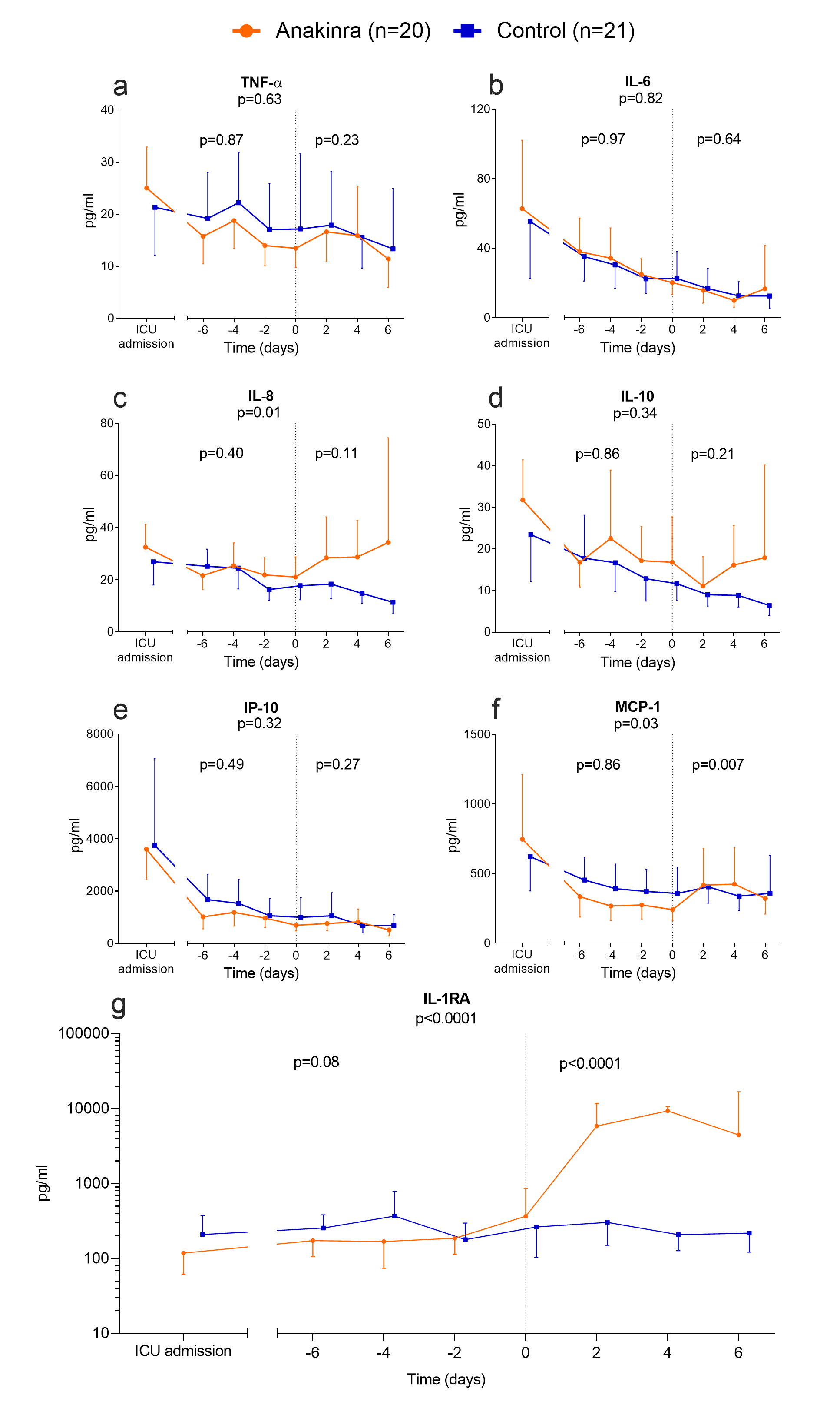

Supplement: Supplementary file 9 — Additional file 9: Figure 3. Description of data: Circulating cytokine concentrations in propensity score-matched groups. Concentrations of circulating (a) tumor necrosis factor (TNF)-α, (b) interleukin (IL)-6, (c) IL-8, (d) IL-10, (e) interferon gamma-induced protein (IP)-10, (f) monocyte chemoattractant protein (MCP)-1, and (g) IL-1 receptor antagonist (IL-1RA) on day of intensive care unit (ICU) admission and serial data within 6 days pre- and post-alignment day (day 0). Data are presented as geometric mean with 95% confidence intervals and were analyzed using mixed-models analysis (time*group interaction factor) to evaluate differences between groups over time. p values under graph titles reflect overall between-group differences (day − 6 until day 6). Between-group p values for day − 6 until day 0 and day 0 until day 6 are shown on the left and right of each panel, respectively. [file 13054_2020_3364_MOESM9_ESM.tif]

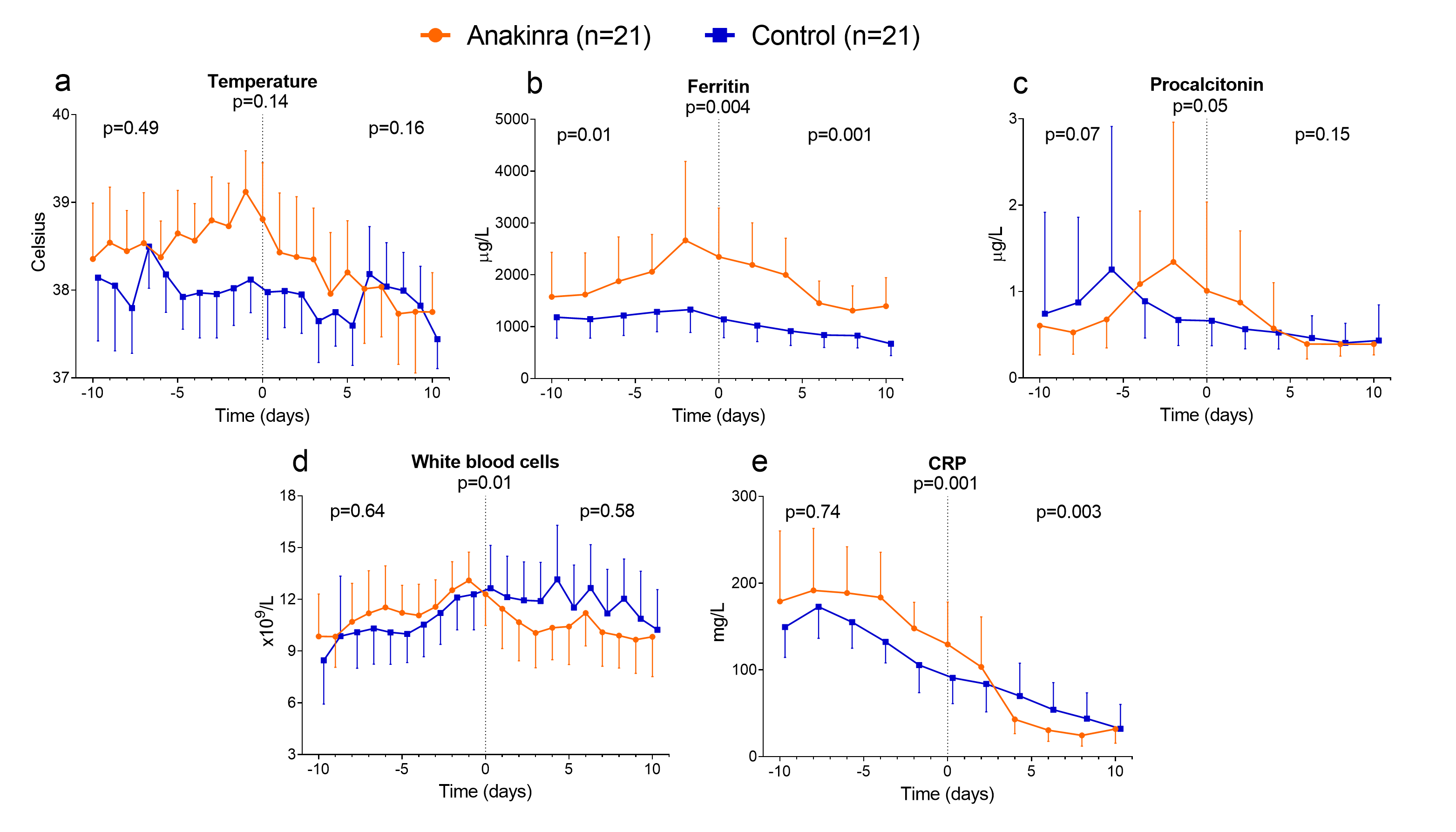

Supplement: Supplementary file 10 — Additional file 10: Figure 4. Description of data: Inflammation parameters over time in propensity score-matched groups. (a) Body temperature and plasma levels of (b) ferritin, (c) procalcitonin, (d) white blood cell counts, and (e) C-reactive protein (CRP) over time within 10 days pre- and post-alignment day (day 0) in propensity scored matched groups (n = 21 both). Data are presented as geometric mean with 95% confidence intervals and were analyzed using mixed-models analysis (time*group interaction factor) to evaluate differences between groups over time. P values under graph titles reflect overall between-group differences (day − 10 until day 10). Between-group p values for day − 10 until day 0 and day 0 until day 10 are shown on the left and right of each panel, respectively. [file 13054_2020_3364_MOESM10_ESM.tif]

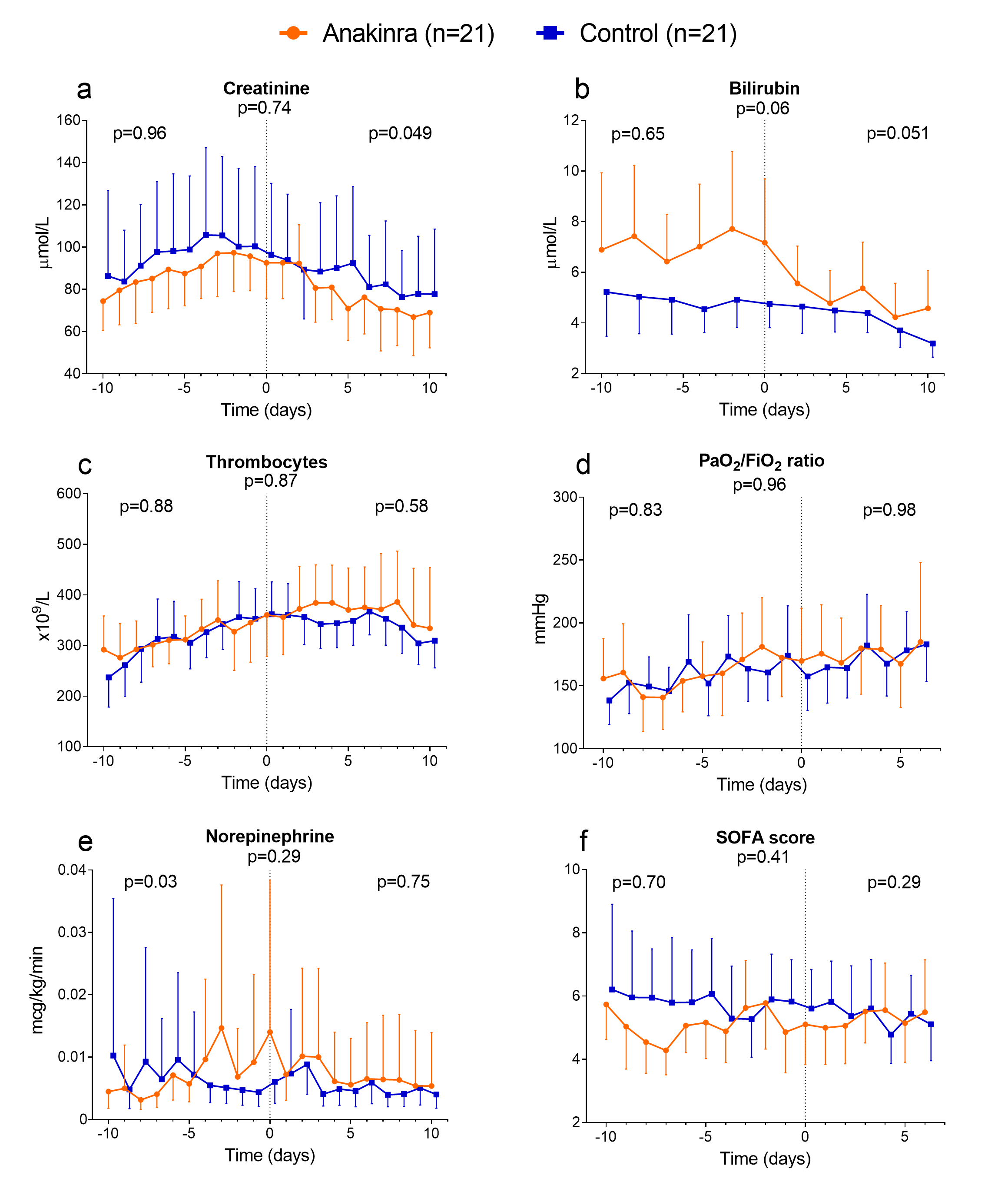

Supplement: Supplementary file 11 — Additional file 11: Figure 5. Description of data: Individual parameters of sequential organ failure assessment (SOFA) score and total SOFA score in propensity score-matched groups. Plasma concentrations of (a) creatinine, (b) bilirubin, and (c) thrombocytes and (d) PaO2/FiO2 (P/F)-ratio, (e) infusion rate of norepinephrine, and (f) SOFA score over time within 10 days pre- and post-alignment day (day 0) in propensity score-matched groups (n = 21 both). PaO2/FiO2 ratio and SOFA score were presented until day 6. Data are presented as geometric mean with 95% confidence intervals and were analyzed using mixed-models analysis (time*group interaction factor) to evaluate differences between groups over time. P values under graph titles reflect overall between-group differences (day − 10 until day 6 or 10). Between-group p values for day − 10 until day 0 and day 0 until day 6 or 10 are shown on the left and right of each panel, respectively. [file 13054_2020_3364_MOESM11_ESM.tif]

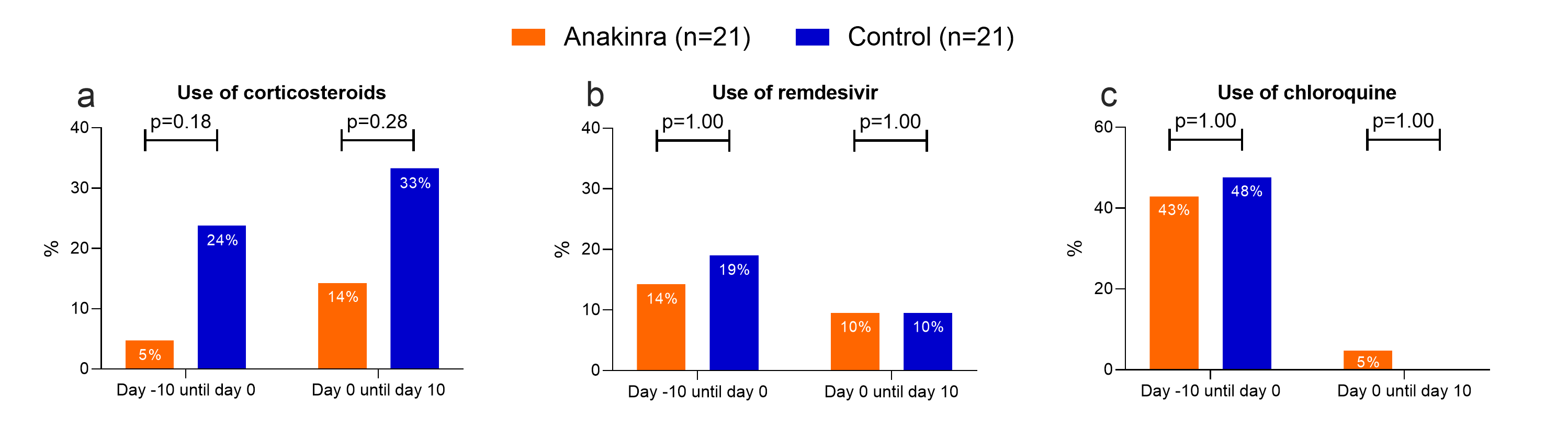

Supplement: Supplementary file 12 — Additional file 12: Figure 6. Description of data: Use of medication in propensity score-matched groups. Differences of use of (a) corticosteroids, (b) remdesivir, and (c) chloroquine between anakinra group and control group during 10 days before and 10 days after alignment day (day 0). p values were calculated using Fisher’s exact tests. [file 13054_2020_3364_MOESM12_ESM.tif]

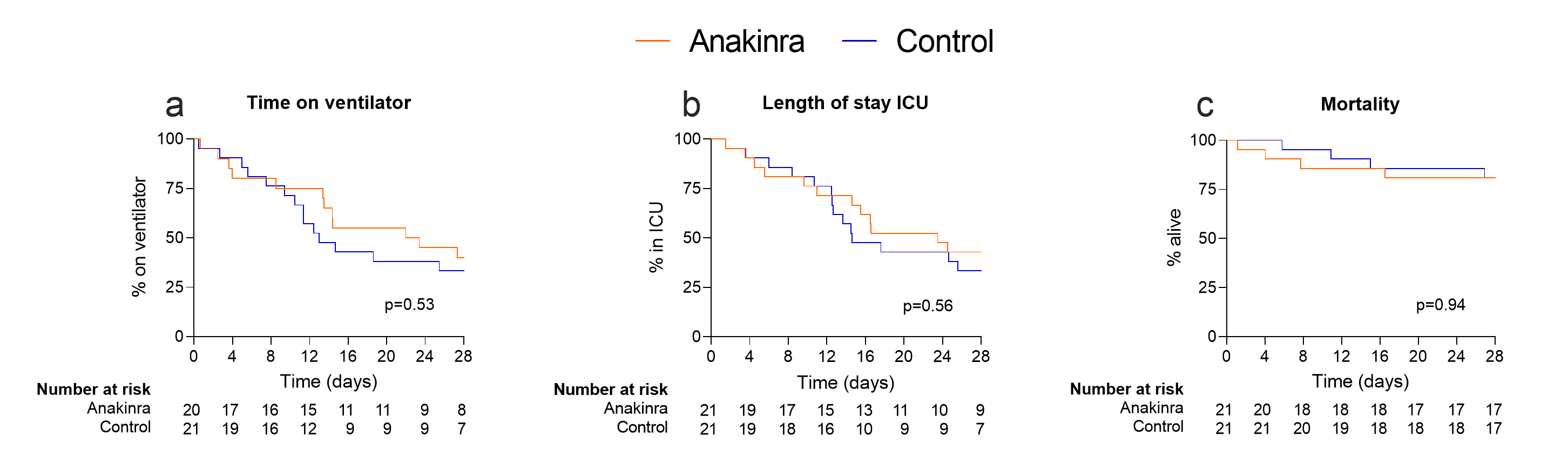

Supplement: Supplementary file 13 — Additional file 13: Figure 7. Description of data: Clinical outcomes in propensity score-matched groups. Kaplan–Meier graphs of (a) time on mechanical ventilator, (b) length of stay in the intensive care unit (ICU), and (c) mortality for propensity score-matched groups. Data are presented for the first 28 days after anakinra alignment day. Patients who were no longer mechanically ventilated on alignment day were not included in time on ventilator graph. P values were calculated using log-rank tests. Numbers at risk on each timepoint per group are shown below graphs. [file 13054_2020_3364_MOESM13_ESM.tif]

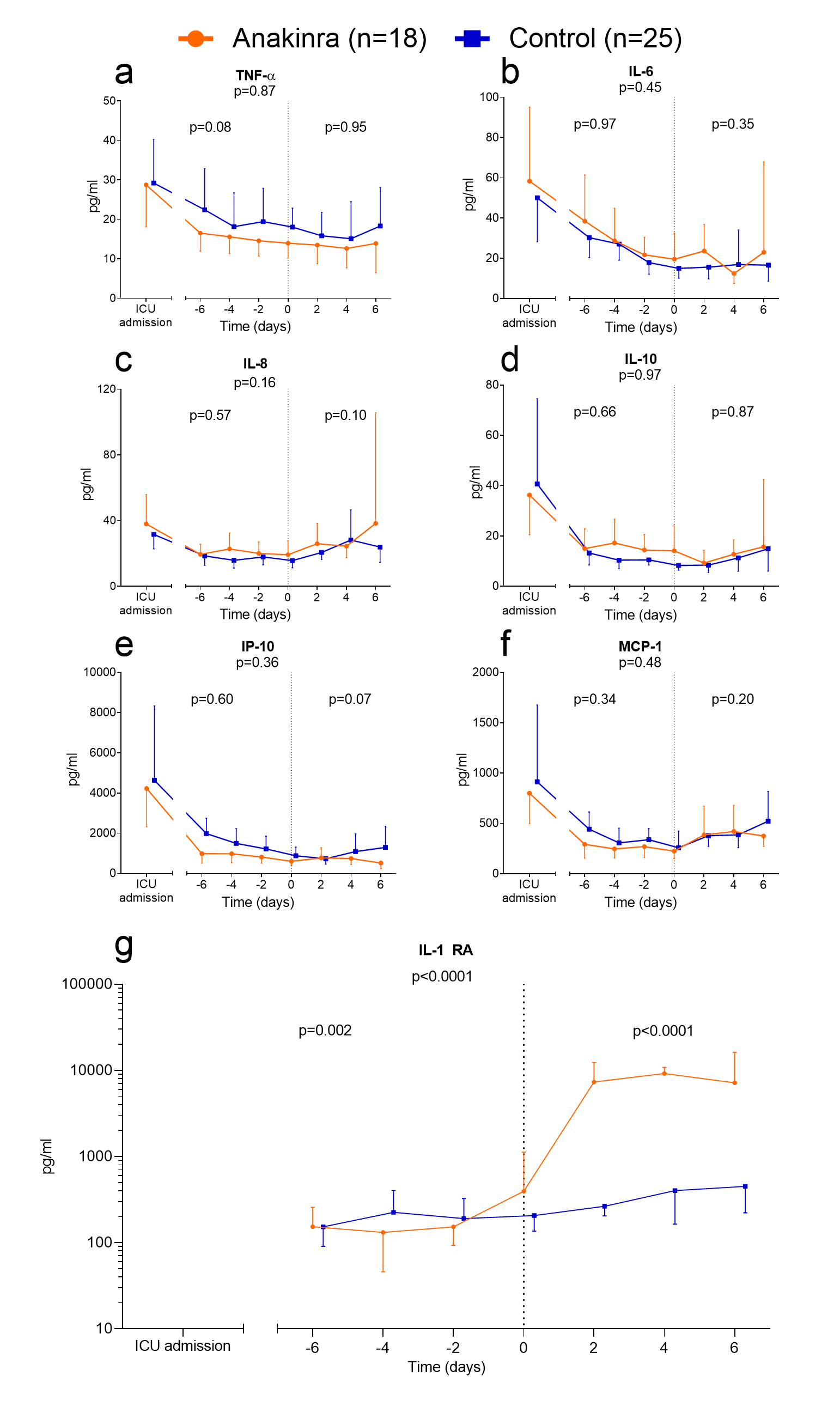

Supplement: Supplementary file 14 — Additional file 14: Figure 8. Description of data: Circulating cytokine concentrations for the subgroup analysis in patients who did not receive corticosteroids. Concentrations of circulating (a) tumor necrosis factor (TNF)-α, (b) interleukin (IL)-6, (c) IL-8, (d) IL-10, (e) interferon gamma-induced protein (IP)-10, (f) monocyte chemoattractant protein (MCP)-1, and (g) IL-1 receptor antagonist (IL-1RA) on day of intensive care unit (ICU) admission and serial data within 6 days pre- and post-alignment day (day 0). Data are presented as geometric mean with 95% confidence intervals and were analyzed using mixed-models analysis (time*group interaction factor) to evaluate differences between groups over time. P values under graph titles reflect overall between-group differences (day − 6 until day 6). Between-group p values for day − 6 until day 0 and day 0 until day 6 are shown on the left and right of each panel, respectively. [file 13054_2020_3364_MOESM14_ESM.tif]

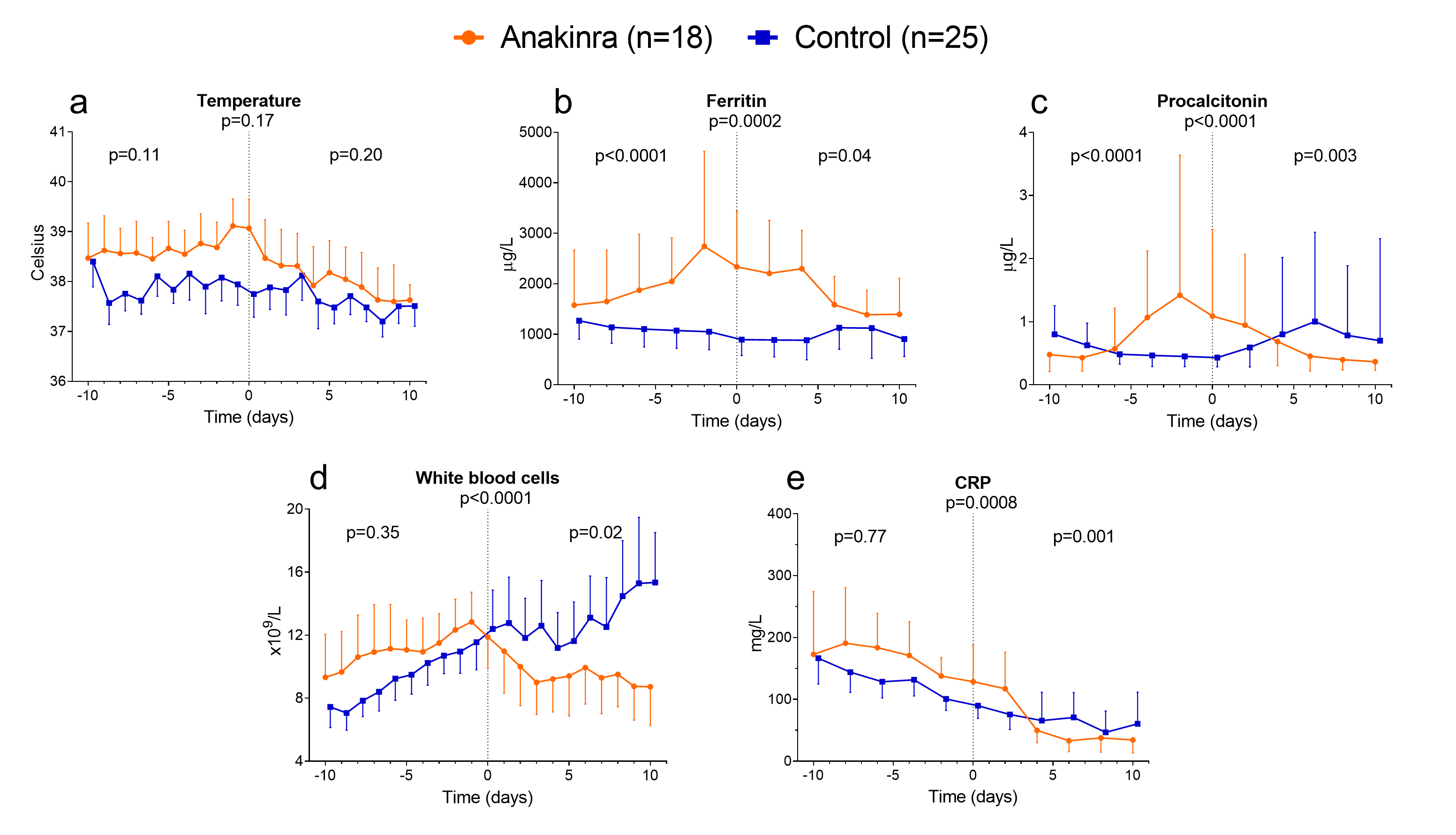

Supplement: Supplementary file 15 — Additional file 15: Figure 9. Description of data: Inflammation parameters over time for the subgroup analysis in patients who did not receive corticosteroids. (a) Body temperature and plasma levels of (b) ferritin, (c) procalcitonin, (d) white blood cell counts, and (e) C-reactive protein (CRP) over time within 10 days pre- and post-alignment day (day 0). Data are presented as geometric mean with 95% confidence intervals and were analyzed using mixed-models analysis (time*group interaction factor) to evaluate differences between groups over time. P values under graph titles reflect overall between-group differences (day − 10 until day 10). Between-group p values for day − 10 until day 0 and day 0 until day 10 are shown on the left and right of each panel, respectively. [file 13054_2020_3364_MOESM15_ESM.tif]

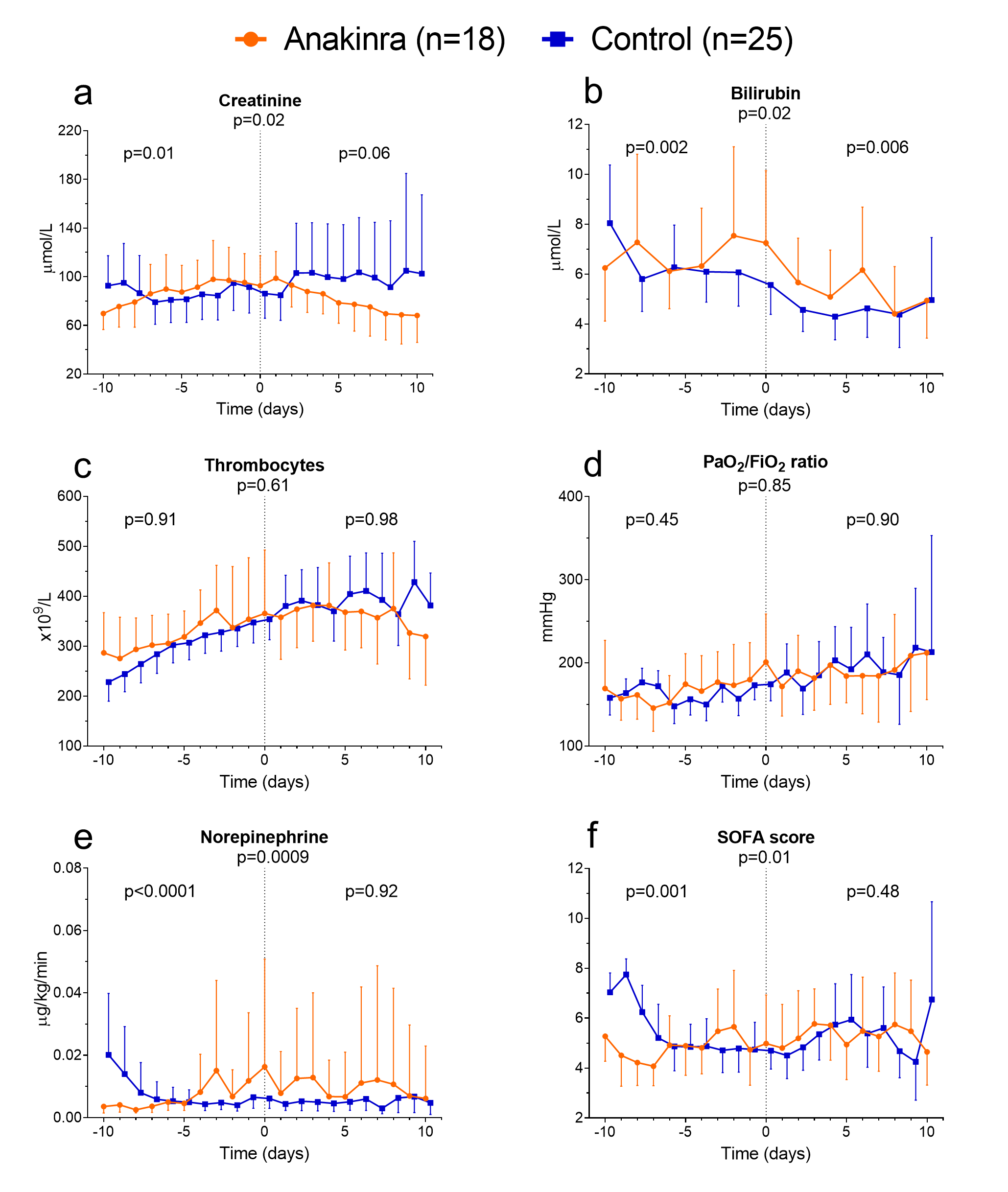

Supplement: Supplementary file 16 — Additional file 16: Figure 10. Description of data: Individual parameters of sequential organ failure assessment (SOFA) score and total SOFA score for the subgroup analysis in patients who did not receive corticosteroids. Plasma concentrations of (a) creatinine, (b) bilirubin, and (c) thrombocytes and (d) PaO2/FiO2 (P/F)-ratio, (e) infusion rate of norepinephrine, and (f) SOFA score over time within 10 days pre- and post-alignment day (day 0). PaO2/FiO2 ratio and SOFA score were presented until day 6. Data are presented as geometric mean with 95% confidence intervals and were analyzed using mixed-models analysis (time*group interaction factor) to evaluate differences between groups over time. P values under graph titles reflect overall between-group differences (day − 10 until day 6 or 10). Between-group p values for day − 10 until day 0 and day 0 until day 6 or 10 are shown on the left and right of each panel, respectively. [file 13054_2020_3364_MOESM16_ESM.tif]

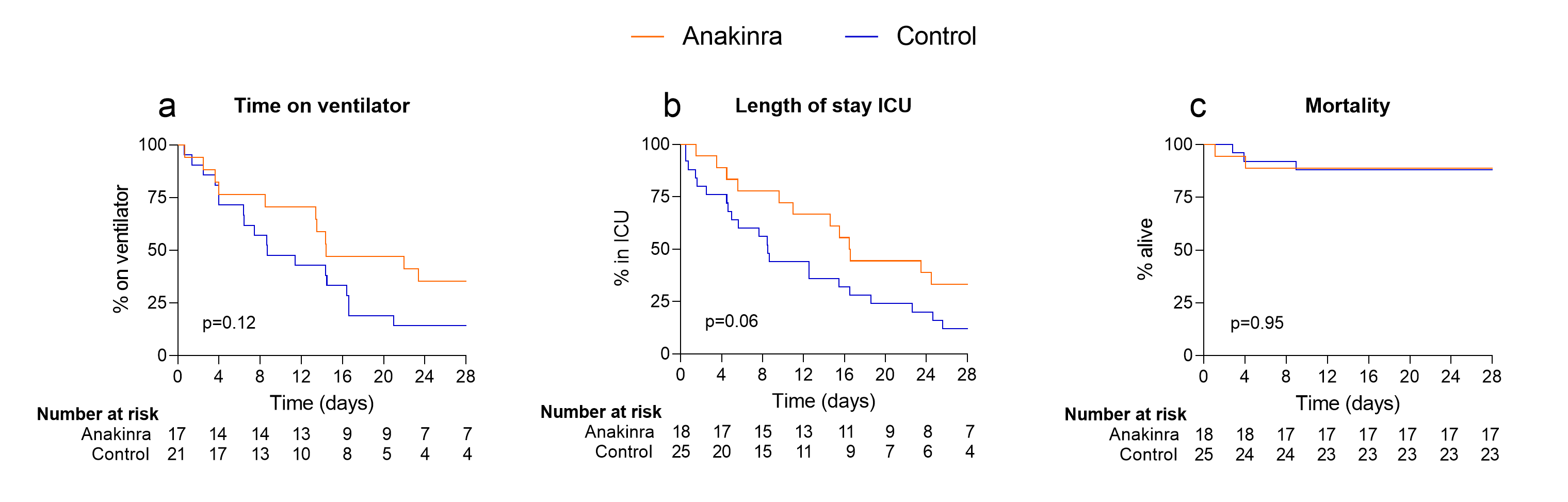

Supplement: Supplementary file 17 — Additional file 17: Figure 11. Description of data: Clinical outcomes for the subgroup analysis in patients who did not receive corticosteroids. Kaplan–Meier graphs of (a) time on mechanical ventilator, (b) length of stay in the intensive care unit (ICU), and (c) mortality. Data are presented for the first 28 days after anakinra alignment day. Patients who were no longer mechanically ventilated on alignment day were not included in time on ventilator graph. p values were calculated using log-rank tests. Numbers at risk on each timepoint per group are shown below graphs. [file 13054_2020_3364_MOESM17_ESM.tif]

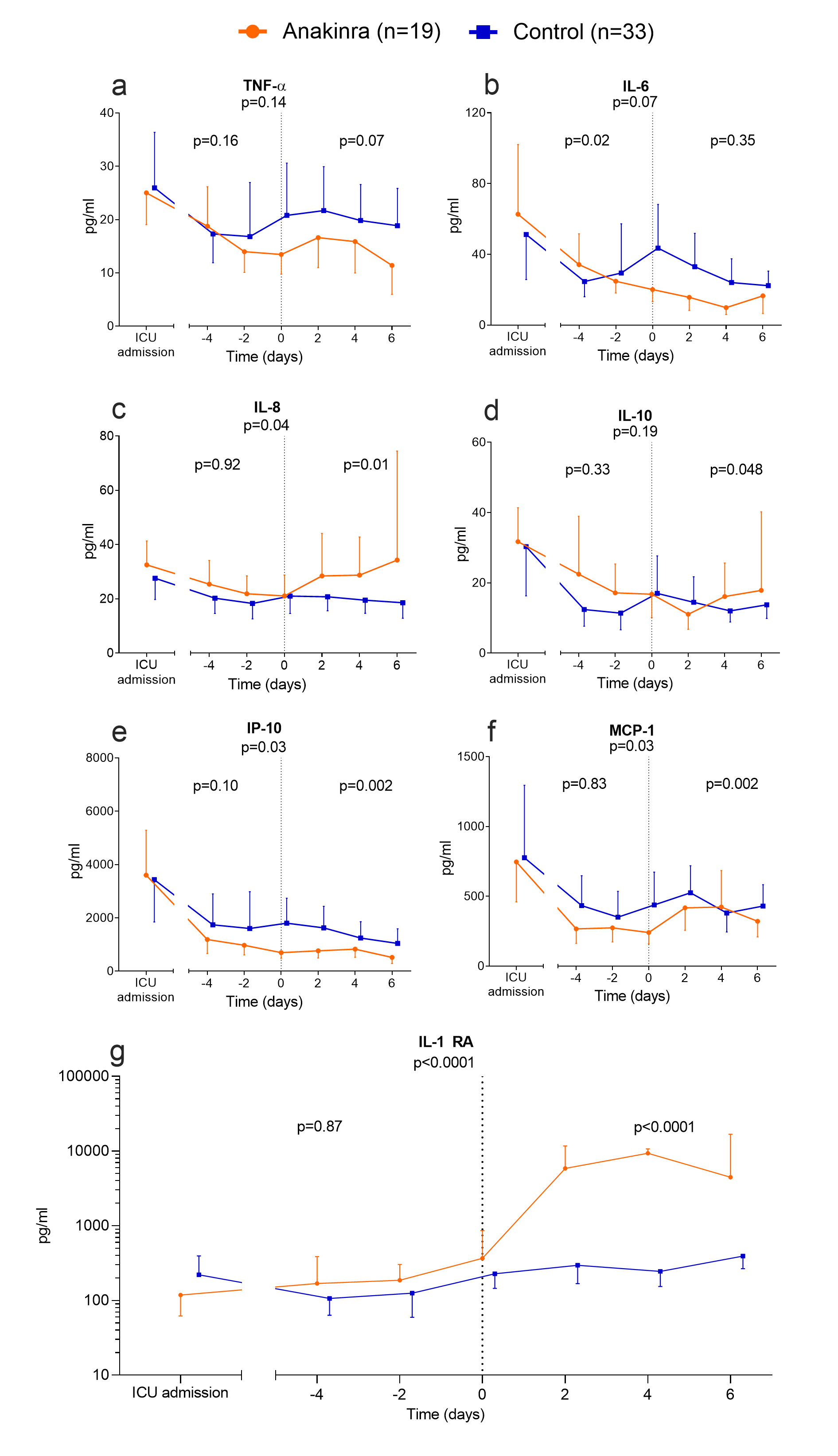

Supplement: Supplementary file 18 — Additional file 18: Figure 12. Description of data: Circulating cytokine concentrations for the subgroup analysis with control patients who partially met the criteria to start anakinra treatment. Concentrations of circulating (a) tumor necrosis factor (TNF)-α, (b) interleukin (IL)-6, (c) IL-8, (d) IL-10, (e) interferon gamma-induced protein (IP)-10, (f) monocyte chemoattractant protein (MCP)-1, and (g) IL-1 receptor antagonist (IL-1RA) on day of intensive care unit (ICU) admission and serial data within four days pre- and 6 days post-alignment day (day 0). Data are presented as geometric mean with 95% confidence intervals and were analyzed using mixed-models analysis (time*group interaction factor) to evaluate differences between groups over time. p values under graph titles reflect overall between-group differences (day − 6 until day 6). Between-group p values for day − 6 until day 0 and day 0 until day 6 are shown on the left and right of each panel, respectively. [file 13054_2020_3364_MOESM18_ESM.tif]

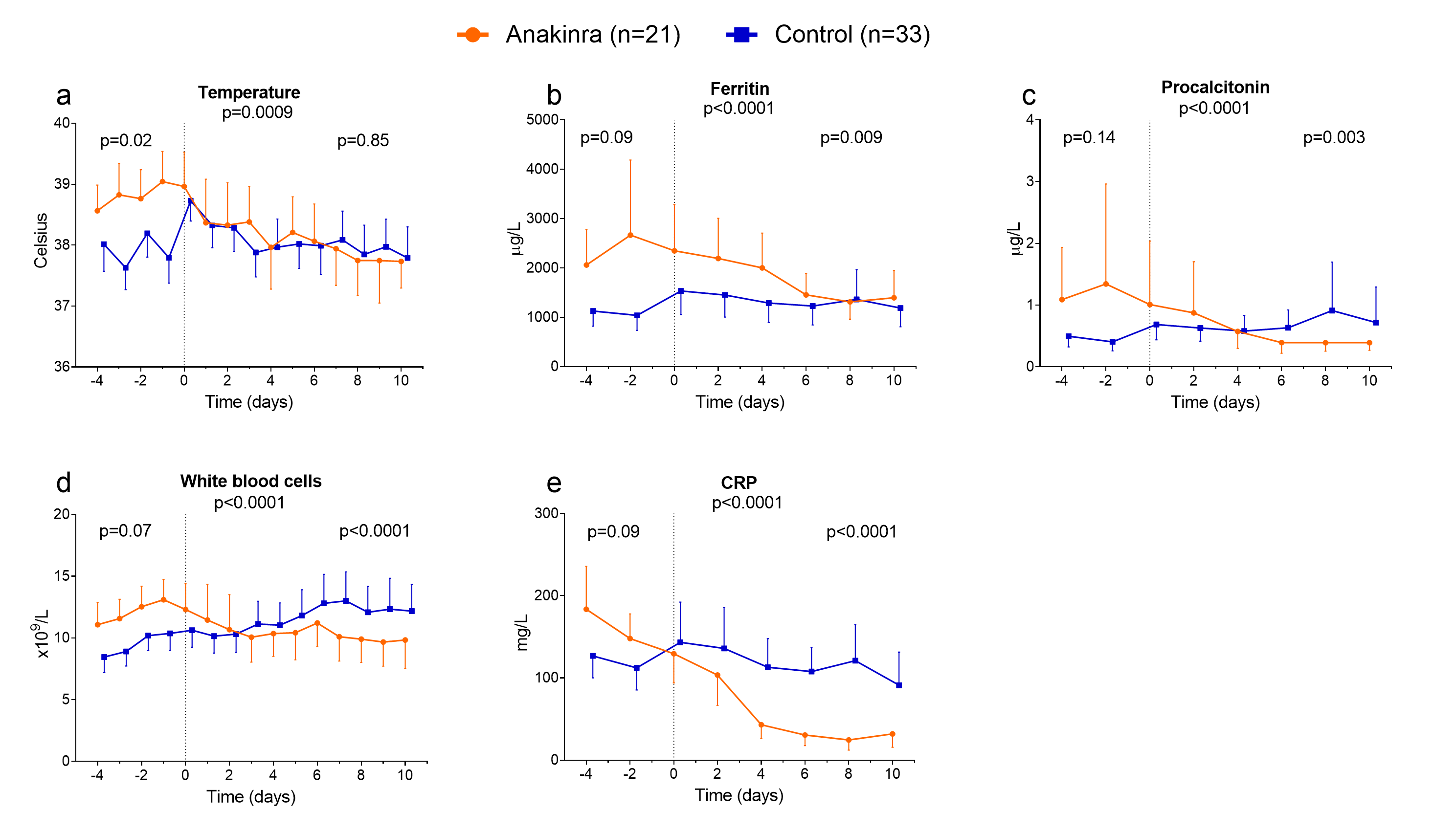

Supplement: Supplementary file 19 — Additional file 19: Figure 13. Description of data: Inflammation parameters over time for the subgroup analysis with control patients who partially met the criteria to start anakinra treatment. (a) Body temperature and plasma levels of (b) ferritin, (c) procalcitonin, (d) white blood cell counts, and (e) C-reactive protein (CRP) over time within 10 days pre- and post-alignment day (day 0). Data are presented as geometric mean with 95% confidence intervals and were analyzed using mixed-models analysis (time*group interaction factor) to evaluate differences between groups over time. P values under graph titles reflect overall between-group differences (day − 10 until day 10). Between-group p values for day − 10 until day 0 and day 0 until day 10 are shown on the left and right of each panel, respectively. [file 13054_2020_3364_MOESM19_ESM.tif]

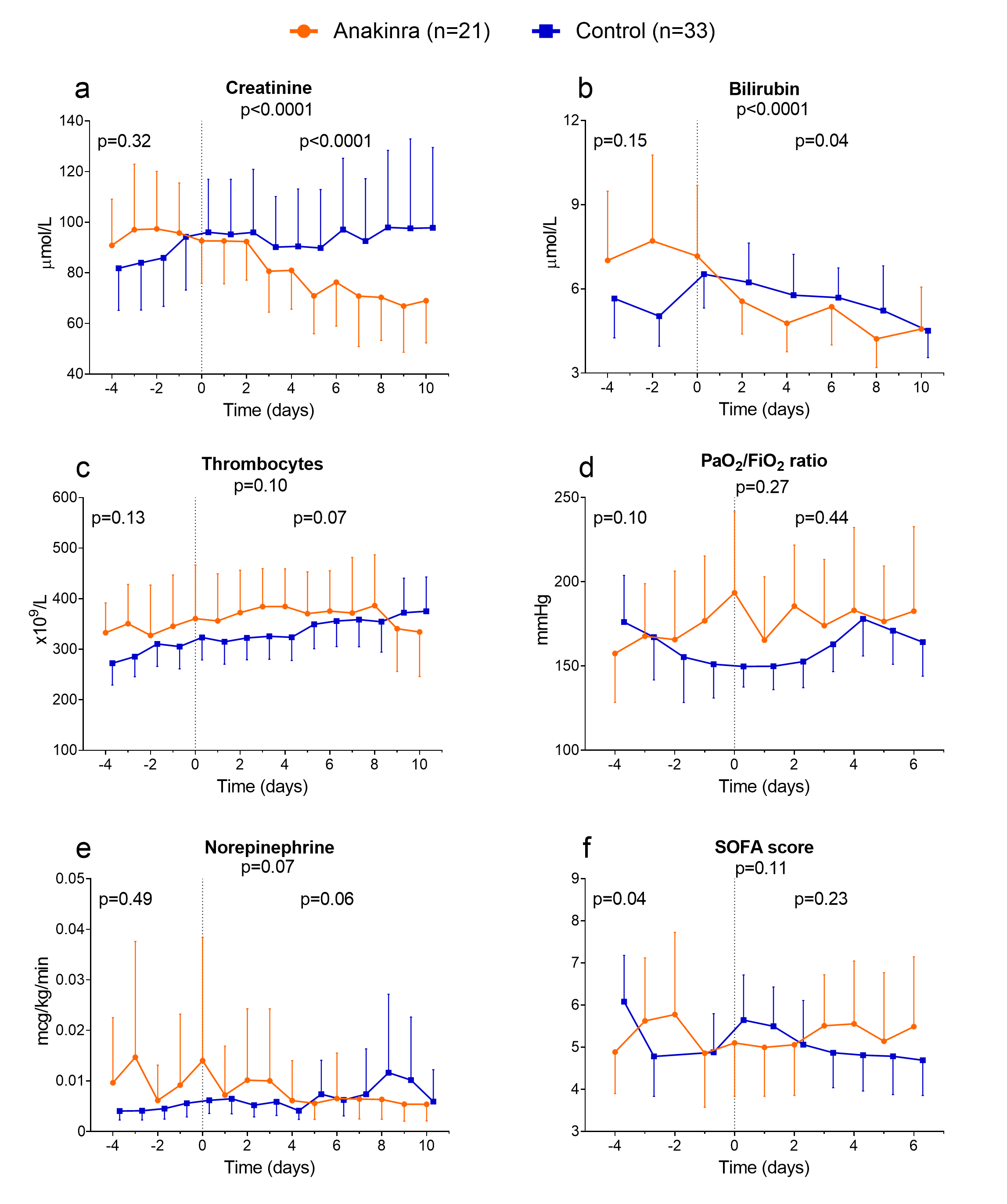

Supplement: Supplementary file 20 — Additional file 20: Figure 14. Description of data: Individual parameters of sequential organ failure assessment (SOFA) score and total SOFA score for the subgroup with control patients who partially met the criteria to start anakinra treatment. Plasma concentrations of (a) creatinine, (b) bilirubin, and (c) thrombocytes and (d) PaO2/FiO2 (P/F)-ratio, (e) infusion rate of norepinephrine, and (f) SOFA score over time within 10 days pre- and post-alignment day (day 0). PaO2/FiO2 ratio and SOFA score were presented until day 6. Data are presented as geometric mean with 95% confidence intervals and were analyzed using mixed-models analysis (time*group interaction factor) to evaluate differences between groups over time. P values under graph titles reflect overall between-group differences (day − 10 until day 6 or 10). Between-group p values for day − 10 until day 0 and day 0 until day 6 or 10 are shown on the left and right of each panel, respectively. [file 13054_2020_3364_MOESM20_ESM.tif]

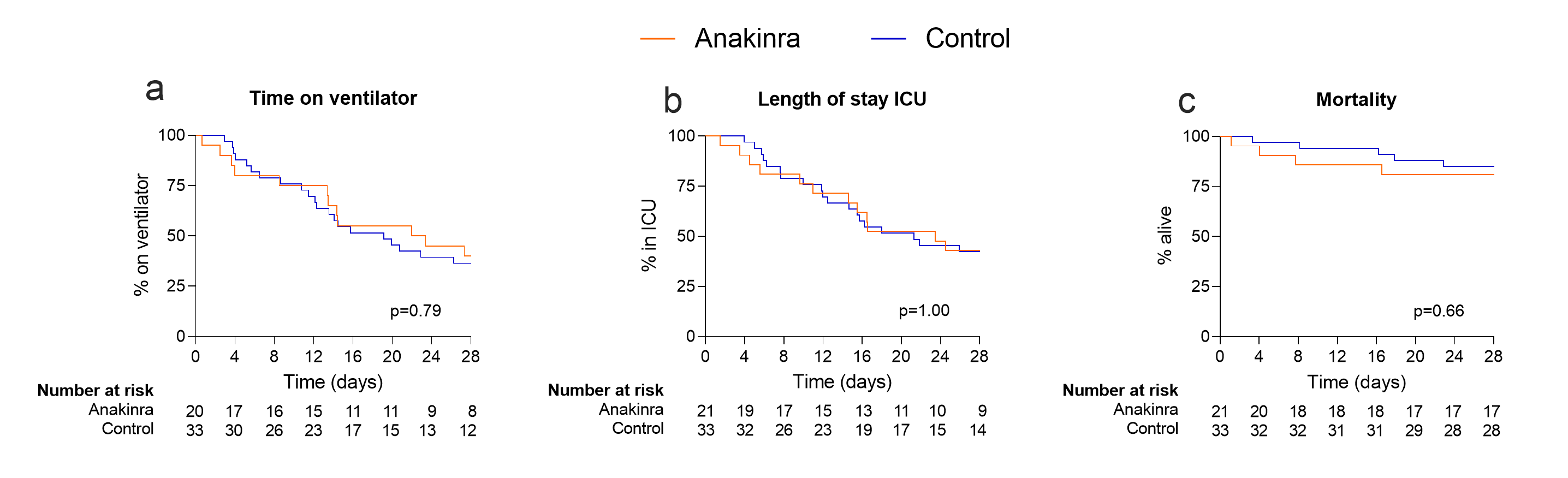

Supplement: Supplementary file 21 — Additional file 21: Figure 15. Description of data: Clinical outcomes for the subgroup with control patients who partially met the criteria to start anakinra treatment. Kaplan–Meier graphs of (a) time on mechanical ventilator, (b) length of stay in the intensive care unit (ICU), and (c) mortality. Data are presented for the first 28 days after anakinra alignment day. Patients who were no longer mechanically ventilated on alignment day were not included in time on ventilator graph. p values were calculated using log-rank tests. Numbers at risk on each timepoint per group are shown below graphs. [file 13054_2020_3364_MOESM21_ESM.tif]
