# Supplementary material for: Anakinra treatment in critically ill COVID-19 patients: a prospective cohort study
Source: Crit Care. 2020 Dec 10;24:688. doi: 10.1186/s13054-020-03364-w (PMC7726611; doi:10.1186/s13054-020-03364-w)
Supplement: Supplementary file 3 — Additional file 3: Table 1. Description of data: Characteristics and clinical parameters at ICU admission and on alignment day for patients included in the inflammatory proteomics analysis. Data are presented as n (%) or median [IQR]. P values were calculated using Fisher’s exact tests and Mann–Whitney U tests. [file 13054_2020_3364_MOESM3_ESM.docx]

**Additional file 3: Supplementary table 1**

**Supplementary table 1.** Characteristics and clinical parameters at ICU admission and on alignment day for patients included in the inflammatory proteomics analysis. Data are presented as n (%) or median [IQR]. P-values were calculated using Fisher’s exact tests and Mann-Whitney-U tests.

|  | Anakinra (n=11) | Control (n=11) | p-value |
| --- | --- | --- | --- |
| Sex, male | 9 (82) | 11 (100) | 0.48 |
| Age, years | 60 [53-73] | 72 [64-73] | 0.19 |
| BMI, kg/m^2^ | 27.2 [25.6-27.8] | 26.7 [24.7-34.0] | 0.90 |
| Apache II | 14 [13-15] | 19 [15-20] | 0.03 |
| Time from first COVID symptoms until ICU admission, days | 13 [10-15] | 10 [7-13] | 0.10 |
| **Medical history** |  |  |  |
| Cardiovascular insufficiency  Hypertension  Respiratory insufficiency  Renal insufficiency  Metastatic neoplasm  Immunological insufficiency  Chronic obstructive pulmonary disease  Diabetes mellitus  Hematologic malignancy | 2 (18)  3 (27)  1 (9)  0 (0)  2 (18)  0 (0)  0 (0)  4 (36)  1 (9) | 7 (64)  8 (73)  0 (0)  1 (9)  1 (9)  1 (9)  1 (9)  3 (27)  0 (0) | 0.08  0.09  1.00  1.00  1.00  1.00  1.00  1.00  1.00 |
| **Clinical parameters on admission day** |  |  |  |
| D-dimer, ng/mL | 3350 [2320-48340] | 2860 [1840-4600] | 0.44 |
| Creatinine, μmol/L | 90 [70-94] | 104 [79-119] | 0.10 |
| Alanine transaminase, U/L | 47 [21-55] | 35 [21-44] | 0.30 |
| Aspartate transaminase, U/L | 44 [37-67] | 53 [33-63] | 0.90 |
| Bilirubin, μmol/L | 8 [6-13] | 7 [6-10] | 0.56 |
| Lactate dehydrogenase, U/L | 380 [310-516] | 388 [359-436] | 0.90 |
| White blood cells, x109/L | 8.2 [6.5-13.4] | 9.1 [5.2 -9.9] | 0.30 |
| Thrombocytes, x109/L | 289 [217-377] | 227 [161-264] | 0.08 |
| C-reactive protein, mg/L | 236 [192-311] | 170 [118-245] | 0.11 |
| Procalcitonin, μg/L | 0.69 [0.30-1.03] | 0.99 [0.26-2.18] | 0.47 |
| Ferritin, μg/L | 1780 [1265-2626] | 1663 [1129-2055] | 0.65 |
| Temperature, °Celsius | 38.6 [38.4-40.0] | 38.6 [37.6-39.5] | 0.80 |
| PaO2/FiO2 ratio, mmHg | 164 [108-206] | 125 [108-158] | 0.37 |
| SOFA score | 7 [4-7] | 6 [4-7] | 0.90 |
| **Clinical parameters on alignment day** |  |  |  |
| D-dimer, ng/mL | 4800 [3909-6840] | 3905 [2450-7190] | 0.56 |
| Creatinine, μmol/L | 92 [79-106] | 128 [91-212] | 0.07 |
| Alanine transaminase, U/L | 91 [48-120] | 51 [38-114] | 0.22 |
| Aspartate transaminase, U/L | 97 [67-132] | 45 [42-89] | 0.008 |
| Bilirubin, μmol/L | 6 [4-7] | 6 [3-11] | 0.70 |
| Lactate dehydrogenase, U/L | 383 [348-450] | 302 [243-375] | 0.03 |
| White blood cells, x109/L | 14.6 [11.5-16.4] | 13.4 [8.5-16.1] | 0.80 |
| Thrombocytes, x109/L | 442 [329-515] | 363 [263-470] | 0.24 |
| C-reactive protein, mg/L | 177 [84-246] | 126 [87-240] | 0.75 |
| Procalcitonin, μg/L | 0.56 [0.34-1.41] | 0.51 [0.41-0.76] | 0.90 |
| Ferritin, μg/L | 2030 [1081-3516] | 1371 [902-2217] | 0.17 |
| Temperature, °Celsius | 39.7 [38.5-40.0] | 37.6 [37.0-38.3] | 0.001 |
| PaO_2_/FiO_2_ ratio, mmHg | 164 [126-268] | 143 [125-212] | 0.75 |
| SOFA score | 5 [5-6] | 5 [4-7] | 0.65 |
| Time from first COVID symptoms until alignment day, days | 22 [19-27] | 21 [18-24] | 0.45 |
