# Supplementary material for: Anakinra treatment in critically ill COVID-19 patients: a prospective cohort study
Source: Crit Care. 2020 Dec 10;24:688. doi: 10.1186/s13054-020-03364-w (PMC7726611; doi:10.1186/s13054-020-03364-w)
Supplement: Supplementary file 5 — Additional file 5. Description of additional results. [file 13054_2020_3364_MOESM5_ESM.docx]

**Additional file 5: Additional results**

**Additional results**
**Propensity score matched analysis**

Propensity score matching increased the similarity in patient characteristics between the anakinra and control groups (Supplementary Table 2, Additional File 6). Analysis of circulating cytokine concentrations showed results comparable to the primary analysis (Supplementary Fig. 3, Additional File 9). Unlike the results of the primary analysis, significance was reached for the decrease in CRP plasma levels in the anakinra group after alignment day (p=0.003) (Supplementary Fig. 4e, Additional File 10). Kinetics of all other clinical parameters were on a par with results of the primary analysis (Supplementary Fig. 4 and 5, Additional Files 10 and 11). No significant differences in use of corticosteroids, remdesivir and chloroquine were present (Supplementary Fig. 6, Additional File 12). Also, analysis of clinical outcomes in propensity score matched groups yielded comparable results (Supplementary Fig. 7, Additional File 13).

**Sensitivity analysis in patients who did not receive corticosteroids**

During the study period, three patients of the anakinra group and 14 patients of the control group were treated with corticosteroids and therefore excluded for this analysis. No significant differences in patient characteristics and clinical parameters on ICU admission day were present (Supplementary Table 3, Additional File 7). Circulating cytokine concentrations showed no significant between-group differences in TNF-α and MCP-1 anymore (Supplementary Fig. 8a, f, Additional File 14). Kinetics of other circulating cytokine concentrations were comparable to those in the primary analysis (Supplementary Fig. 8, Additional File 14). The clinical inflammatory parameters showed largely comparable results to the primary analysis (Supplementary Fig. 9, Additional File 15), with the decrease in CRP after start of anakinra treatment becoming significant (p=0.001), whereas significance was lost for temperature. The decrease in creatinine after start of anakinra treatment remained present, albeit slightly less pronounced than in the primary analysis (p=0.06, Supplementary Fig. 10a, Additional File 16). Kinetics of bilirubin plasma levels, thrombocyte counts, PaO2/FiO2 ratio, norepinephrine infusion rate and total SOFA score were comparable to the results in our primary analysis (Supplementary Fig. 10, Additional File 16). As in the primary analysis, no differences in time on mechanical ventilation, ICU length of stay and mortality were present (Supplementary Fig. 11, Additional File 17).

**Sensitivity analysis using control patients with persisting fever/high ferritin plasma levels**

A total of 33 patients of the control group of the main analysis developed a period of fever (n=17) or high ferritin plasma levels (n=16) during their ICU stay. The median alignment day of this subgroup was day 6 post-ICU admission. No significant differences in patient characteristics and clinical parameters on ICU admission were present between this control group and the anakinra group (Supplementary Table 4, Additional File 8). On alignment day, PaO_2_/FiO_2_ ratio was 188 [133-268] mmHg in the anakinra group versus 155 [124-170] mmHg in the control group (p=0.03). The time between the start of COVID-19 symptoms and alignment day was 22 [19-27] days in the anakinra group and 18 [13-23] days in the control group (p=0.008). No other significant differences between both groups were present on alignment day (Supplementary Table 4, Additional File 8). In general, effects of anakinra treatment on inflammatory parameters in this sensitivity analysis were comparable to those of the main analysis, although statistical significance was lost for some parameters (temperature) and emerged for others (CRP, several cytokines, Supplementary Fig. 12-13, Additional Files 18-19). The significant between-group differences in several cytokines in this sensitivity analysis are likely due to the earlier day of alignment of the control group (day 6 vs. day 12 in the main analyses). As cytokine levels are highest on ICU admission and gradually decrease thereafter (see Fig. 1), the 6-day difference in day of alignment is a plausible explanation for the higher concentrations of circulating cytokines on alignment day in the control group (Supplementary Fig. 12, Additional File 18). Results of other clinical inflammatory parameters, SOFA-score (and its individual components) and clinical outcomes were comparable to the results of the main analysis (Supplementary Fig. 13-15, Additional Files 19-21).
