# Supplementary material for: Anakinra treatment in critically ill COVID-19 patients: a prospective cohort study
Source: Crit Care. 2020 Dec 10;24:688. doi: 10.1186/s13054-020-03364-w (PMC7726611; doi:10.1186/s13054-020-03364-w)
Supplement: Supplementary file 6 — Additional file 6: Table 2. Description of data: Patient characteristics and clinical parameters at ICU admission and on alignment day in propensity score-matched groups. Data are presented as n (%) or median [IQR]. P values were calculated using Fisher’s exact tests and Mann–Whitney U tests. [file 13054_2020_3364_MOESM6_ESM.docx]

**Additional file 6: Supplementary table 2**

**Supplementary table 2.** Patient characteristics and clinical parameters at ICU admission and on alignment day in propensity score matched groups. Data are presented as n (%) or median [IQR]. P-values were calculated using Fisher’s exact tests and Mann-Whitney-U tests.

|  | Anakinra (n=21) | Control (n=21) | p-value |
| --- | --- | --- | --- |
| Sex, male | 14 (67) | 15 (71) | 1.00 |
| Age, years | 63 [55-71] | 65 [61-72] | 0.50 |
| BMI, kg/m2 | 27.7 [25.9-29.9] | 25.7 [24.2-28.2] | 0.13 |
| Apache II | 15 [13-18] | 15 [12-19] | 0.74 |
| Days first COVID symptoms until ICU admission, days | 13 [9-14] | 11 [8-15] | 0.67 |
| **Medical history** |  |  |  |
| Cardiovascular insufficiency  Hypertension  Respiratory insufficiency  Renal insufficiency  Metastatic neoplasm  Immunological insufficiency  Chronic obstructive pulmonary disease  Diabetes mellitus  Hematologic malignancy | 4 (19)  8 (38)  1 (5)  0 (0)  2 (10)  0 (0)  0 (0)  7 (33)  1 (5) | 8 (38)  11 (52)  2 (10)  0 (0)  2 (10)  1 (5)  1 (5)  4 (19)  0 (0) | 0.31  0.54  1.00  1.00  1.00  1.00  1.00  0.48  1.00 |
| **Clinical parameters on admission day** |  |  |  |
| D-dimer, ng/mL | 3380 [2028-18343] | 2950 [1835-5765] | 0.38 |
| Creatinine, μmol/L | 84 [68-96] | 94 [57-111] | 0.80 |
| Alanine transaminase, U/L | 47 [21-62] | 40 [28-57] | 0.90 |
| Aspartate transaminase, U/L | 55 [37-76] | 52 [37-63] | 0.89 |
| Bilirubin, μmol/L | 8 [6-13] | 7 [5-10] | 0.28 |
| Lactate dehydrogenase, U/L | 380 [322-493] | 388 [324-469] | 0.82 |
| White blood cells, x10^9^/L | 8.2 [7.0-12.0] | 9.1 [6.3-11.7] | 0.86 |
| Thrombocytes, x10^9^/L | 247 [189-324] | 263 [168-344] | 0.07 |
| C-reactive protein, mg/L | 254 [188-297] | 196 [158-296] | 0.40 |
| Procalcitonin, μg/L | 0.66 [0.18-1.39] | 1.11 [0.41-4.05] | 0.19 |
| Ferritin, μg/L | 1842 [1313-2767] | 1452 [1026-1879] | 0.11 |
| Temperature, °Celsius | 38.4 [37.8-38.9] | 38.3 [37.7-39.7] | 0.71 |
| PaO_2_/FiO_2_ ratio, mmHg | 138 [105-199] | 125 [103-182] | 0.77 |
| SOFA score | 7 [4-7] | 6 [5-7] | 0.56 |
| **Clinical parameters on alignment day** |  |  |  |
| D-dimer, ng/mL | 4063 [2585-6285] | 4730 [2970-6855] | 0.51 |
| Creatinine, μmol/L | 92 [76-106] | 100 [55-154] | 0.85 |
| Alanine transaminase, U/L | 89 [55-119] | 83 [54-208] | 0.95 |
| Aspartate transaminase, U/L | 96 [67-142] | 77 [46-122] | 0.23 |
| Bilirubin, μmol/L | 6 [5-12] | 4 [3-6] | 0.04 |
| Lactate dehydrogenase, U/L | 371 [317-450] | 358 [276-403] | 0.26 |
| White blood cells, x10^9^/L | 12.4 [10.4-15.9] | 12.9 [10.2-16.1] | 0.97 |
| Thrombocytes, x10^9^/L | 351 [314-494] | 391 [280-468] | 0.40 |
| C-reactive protein, mg/L | 130 [90-237] | 97 [46-173] | 0.16 |
| Procalcitonin, μg/L | 0.66 [0.39-1.88] | 0.62 [0.24-1.38] | 0.42 |
| Ferritin, μg/L | 2365 [1272-3713] | 1123 [805-2011] | 0.005 |
| Temperature, °Celsius | 39.1 [38.3-40.0] | 37.8 [37.2-38.8] | 0.009 |
| PaO_2_/FiO_2_ ratio, mmHg | 188 [133-268] | 163 [120-255 | 0.41 |
| SOFA score | 6 [4-8] | 6 [4-8] | 0.78 |
| Time from first COVID symptoms until alignment day, days | 22 [19-27] | 23 [18-27] | 0.84 |
