# Supplementary material for: Anakinra treatment in critically ill COVID-19 patients: a prospective cohort study
Source: Crit Care. 2020 Dec 10;24:688. doi: 10.1186/s13054-020-03364-w (PMC7726611; doi:10.1186/s13054-020-03364-w)
Supplement: Supplementary file 7 — Additional file 7: Table 3. Description of data: Patient characteristics and clinical parameters at ICU admission and on alignment day for the subgroup analysis in patients who received no corticosteroids. Data are presented as n (%) or median [IQR]. P values were calculated using Fisher’s exact tests and Mann–Whitney U tests. [file 13054_2020_3364_MOESM7_ESM.docx]

**Additional file 7: Supplementary table 3**

**Supplementary table 3.** Patient characteristics and clinical parameters at ICU admission and on alignment day for the subgroup analysis in patients who received no corticosteroids. Data are presented as n (%) or median [IQR]. P-values were calculated using Fisher’s exact tests and Mann-Whitney-U tests.

|  | Anakinra (n=18) | Control (n=25) | p-value |
| --- | --- | --- | --- |
| Sex, male | 12 (67) | 21 (84) | 0.28 |
| Age, years | 60 [52-68] | 65 [57-71] | 0.44 |
| BMI, kg/m2 | 27.7 [25.4-31.0] | 27.4 [24.1-31.1] | 0.49 |
| Apache II | 15 [13-18] | 15 [11-20] | 0.68 |
| Time from first COVID symptoms until ICU admission, days | 13 [8-14] | 10 [7-15] | 0.42 |
| **Medical history** |  |  |  |
| Cardiovascular insufficiency  Hypertension  Respiratory insufficiency  Renal insufficiency  Metastatic neoplasm  Immunological insufficiency  COPD  Diabetes mellitus  Hematologic malignancy | 3 (0.17)  6 (33)  1 (6)  0 (0)  2 (11)  0 (0)  0 (0)  6 (33)  1 (6) | 6 (24)  15 (60)  2 (8)  0 (0)  1 (4)  1 (4)  3 (12)  5 (20)  0 (0) | 0.71  0.12  1.00  1.00  0.56  1.00  0.25  0.48  0.42 |
| **Clinical parameters on admission day** |  |  |  |
| D-dimer, ng/mL | 3410 [1905-33450] | 2905 [1592-4388] | 0.18 |
| Creatinine, μmol/L | 91 [69-100] | 82 [63-111] | 0.89 |
| Alanine transaminase, U/L | 48 [21-58] | 40 [31-61] | 0.76 |
| Aspartate transaminase, U/L | 50 [36-79] | 51 [38-63] | 0.94 |
| Bilirubin, μmol/L | 8 [6-14] | 7 [5-13] | 0.69 |
| Lactate dehydrogenase, U/L | 367 [318-506] | 421 [333-523] | 0.71 |
| White blood cells, x10^9^/L | 8.2 [6.5-10.5] | 9.3 [5.6-11.0] | 0.99 |
| Thrombocytes, x10^9^/L | 240 [197-313] | 227 [176-314] | 0.61 |
| C-reactive protein, mg/L | 254 [190-295] | 186 [120-292] | 0.12 |
| Procalcitonin, μg/L | 0.69 [0.19-1.37] | 0.74 [0.33-1.98] | 0.81 |
| Ferritin, μg/L | 1695 [1210-2985] | 1311 [556-2535] | 0.13 |
| Temperature, °Celsius | 38.5 [37.7-39.0] | 38.5 [37.5-39.2] | 0.82 |
| PaO_2_/FiO_2_ ratio, mmHg | 143 [103-209] | 143 [103-179] | 0.89 |
| SOFA score | 7 [5-7] | 7 [5-8] | 0.13 |
| **Clinical parameters on alignment day** |  |  |  |
| D-dimer, ng/mL | 4063 [2775-6055] | 3935 [3160-5900] | 0.91 |
| Creatinine, μmol/L | 93 [73-118] | 72 [46-148] | 0.51 |
| Alanine transaminase, U/L | 91 [65-118] | 67 [51-107] | 0.23 |
| Aspartate transaminase, U/L | 97 [67-139] | 64 [48-97] | 0.02 |
| Bilirubin, μmol/L | 6 [5-12] | 5 [4-9] | 0.21 |
| Lactate dehydrogenase, U/L | 383 [318-450] | 349 [258-425] | 0.22 |
| White blood cells, x10^9^/L | 11.9 [10.3-16.7] | 13.3 [9.2-16.1] | 0.61 |
| Thrombocytes, x10^9^/L | 379 [319-497] | 376 [263-431] | 0.25 |
| C-reactive protein, mg/L | 149 [76-248] | 87 [59-145] | 0.07 |
| Procalcitonin, μg/L | 0.46 [0.30-0.68] | 0.58 [0.29-0.83] | 0.42 |
| Ferritin, μg/L | 2344 [1353-4397] | 1247 [447-1680] | 0.002 |
| Temperature, °Celsius | 39.2 [38.3-40.0] | 37.8 [37.0-38.5] | 0.001 |
| PaO_2_/FiO_2_ ratio, mmHg | 206 [136-275] | 157 [143-231] | 0.24 |
| SOFA score | 6 [4-8] | 5 [4-7] | 0.28 |
| Time from first COVID symptoms until alignment day, days | 22 [19-26] | 21 [18-26] | 0.92 |
