# Supplementary material for: Anakinra treatment in critically ill COVID-19 patients: a prospective cohort study
Source: Crit Care. 2020 Dec 10;24:688. doi: 10.1186/s13054-020-03364-w (PMC7726611; doi:10.1186/s13054-020-03364-w)
Supplement: Supplementary file 8 — Additional file 8: Table 4. Description of data: Patient characteristics and clinical parameters at ICU admission and on alignment day for the subgroup analysis with control patients who partially met the criteria to start anakinra treatment. Data are presented as n (%) or median [IQR]. P values were calculated using Fisher’s exact tests and Mann–Whitney U tests. [file 13054_2020_3364_MOESM8_ESM.docx]

**Additional file 8: Supplementary table 4**

**Supplementary table 4.** Patient characteristics and clinical parameters at ICU admission and on alignment day for the subgroup analysis with control patients who partially met the criteria to start anakinra treatment. Data are presented as n (%) or median [IQR]. P-values were calculated using Fisher’s exact tests and Mann-Whitney-U tests.

|  | Anakinra (n=21) | Control (n=33) | p-value |
| --- | --- | --- | --- |
| Sex, male | 14 | 28 | 0.18 |
| Age, years | 63 [55-71] | 67 [59-72] | 0.46 |
| BMI, kg/m2 | 27.7 [25.9-29.9] | 25.8 [24.2-29.5] | 0.30 |
| Apache II | 15 [13-18] | 16 [12-20] | 0.51 |
| Days first COVID symptoms until ICU admission, days | 13 [9-14] | 10 [7-16] | 0.38 |
| **Medical history** |  |  |  |
| Cardiovascular insufficiency  Hypertension  Respiratory insufficiency  Renal insufficiency  Metastatic neoplasm  Immunological insufficiency  Chronic obstructive pulmonary disease  Diabetes mellitus  Hematologic malignancy | 4 (19)  8 (38)  1 (5)  0 (0)  2 (10)  0 (0)  0 (0)  7 (33)  1 (5)1 | 7 (21)  20 (61)  1 (3)  0 (0)  2 (6)  1 (3)  3 (9)  7 (21)  0 (0) | 1.00  0.16  1.00  1.00  0.64  1.00  0.27  0.36  0.39 |
| **Clinical parameters on admission day** |  |  |  |
| D-dimer, ng/mL | 3380 [2028-18343] | 2905 [1668-4568] | 0.24 |
| Creatinine, μmol/L | 84 [68-96] | 82 [70-110] | 0.67 |
| Alanine transaminase, U/L | 47 [21-62] | 39 [30-62] | 0.88 |
| Aspartate transaminase, U/L | 55 [35-76] | 51 [41-63] | 0.91 |
| Bilirubin, μmol/L | 8 [6-13] | 7 [6-13] | 0.53 |
| Lactate dehydrogenase, U/L | 380 [322-493] | 414 [324-488] | 0.98 |
| White blood cells, x10^9^/L | 8.2 [7.0-12.0] | 9.8 [6.5-11.2] | 0.89 |
| Thrombocytes, x10^9^/L | 247 [189-324] | 244 [175-325] | 0.77 |
| C-reactive protein, mg/L | 254 [188-297] | 210 [167-292] | 0.33 |
| Procalcitonin, μg/L | 0.66 [0.18-1.39] | 0.87 [0.39-2.95] | 0.37 |
| Ferritin, μg/L | 1842 [1313-2767] | 1469 [879-2457] | 0.31 |
| Temperature, °Celsius | 38.4 [37.8-38.9] | 38.9 [37.7-39.8] | 0.35 |
| PaO_2_/FiO_2_ ratio, mmHg | 138 [105-199] | 139 [93-178] | 0.84 |
| SOFA score | 7 [4-7] | 6 [5-8] | 0.38 |
| **Clinical parameters on alignment day** |  |  |  |
| D-dimer, ng/mL | 4063 [2585-6285] | 3580 [2545-6380] | 0.96 |
| Creatinine, μmol/L | 92 [76-106] | 80 [62-153] | 0.65 |
| Alanine transaminase, U/L | 89 [55-119] | 78 [38-187] | 0.74 |
| Aspartate transaminase, U/L | 96 [67-142] | 75 [57-119] | 0.40 |
| Bilirubin, μmol/L | 6 [5-12] | 6 [4-10] | 0.68 |
| Lactate dehydrogenase, U/L | 371 [317-450] | 383 [301-479] | 0.82 |
| White blood cells, x10^9^/L | 12.4 [10.4-15.9] | 11.5 [9.1-13.4] | 0.09 |
| Thrombocytes, x10^9^/L | 351 [314-494] | 320 [224-426] | 0.11 |
| C-reactive protein, mg/L | 130 [90-237] | 185 [85-283] | 0.52 |
| Procalcitonin, μg/L | 0.48 [0.33-0.69] | 0.66 [0.24-1.32] | 0.62 |
| Ferritin, μg/L | 2365 [1272-3713] | 1929 [654-3287] | 0.13 |
| Temperature, °Celsius | 39.1 [38.3-40.0] | 38.7 [38.3-39.4] | 0.32 |
| PaO_2_/FiO_2_ ratio, mmHg | 188 [133-268] | 155 [124-170] | **0.03** |
| SOFA score | 6 [4-8] | 6 [4-8] | 0.59 |
| Time from first COVID symptoms until alignment day, days | 22 [19-27] | 18 [13-23] | **0.008** |
